# Discovery of Novel Pterostilbene Derivatives That Might Treat Sepsis by Attenuating Oxidative Stress and Inflammation through Modulation of MAPKs/NF-κB Signaling Pathways

**DOI:** 10.3390/antiox10091333

**Published:** 2021-08-24

**Authors:** Mengyuan Fang, Tingfeng Zou, Xiaoxiao Yang, Zhen Zhang, Peichang Cao, Jihong Han, Yajun Duan, Ban-Feng Ruan, Qing-Shan Li

**Affiliations:** 1 Key Laboratory of Metabolism and Regulation for Major Diseases of Anhui Higher Education Institutes, College of Food and Biological Engineering, Hefei University of Technology, Hefei 230601, China; 2019171303@mail.hfut.edu.cn (M.F.); 2020171463@mail.hfut.edu.cn (T.Z.); 2019171310@mail.hfut.edu.cn (Z.Z.); 2018171113@mail.hfut.edu.cn (P.C.); hanjihong2015@hfut.edu.cn (J.H.); yduan@hfut.edu.cn (Y.D.); 2 Key Lab of Biofabrication of Anhui Higher Education Institution Centre for Advanced Biofabrication, Hefei University, Hefei 230601, China

**Keywords:** antioxidants, anti-inflammatory agents, indanone, pterostilbene, sepsis

## Abstract

Sepsis remains one of the most common life-threatening illnesses that is characterized by a systemic inflammatory response syndrome (SIRS) and usually arises following severe trauma and various septic infections. It is still in urgent need of new effective therapeutic agents, and chances are great that some candidates can be identified that can attenuate oxidative stress and inflammatory responses. Pterostilbene, which exerts attractive anti-oxidative and anti-inflammatory activities, is a homologue of natural polyphenolic derivative of resveratrol. Starting from it, we have made several rounds of rational optimizations. Firstly, based on the strategy of pharmacophore combination, indanone moiety was introduced onto the pterostilbene skeleton to generate a novel series of pterostilbene derivatives (**PIF_1**–**PIF_16**) which could possess both anti-oxidative and anti-inflammatory activities for sepsis treatment. Then, all target compounds were subjected to their structure–activity relationships (SAR) screening of anti-inflammatory activity in mouse mononuclear macrophage RAW264.7 cell line, and their cytotoxicities were determined after. Finally, an optimal compound, **PIF_9**, was identified. It decreased the mRNA levels of lipopolysaccharide (LPS)-induced interleukin-1β (IL-1β), tumor necrosis factor-α (TNF-α), inducible nitric oxide synthase (iNOS), and cyclooxygenase 2 (COX2). We also found that the anti-inflammatory effects might be contributed by its suppression on the nuclear factor-κB (NF-κB) and MAPKs signaling pathway. Moreover, **PIF_9** also demonstrated potent anti-oxidative activity in RAW264.7 macrophages and the sepsis mouse model. Not surprisingly, with the benefits mentioned above, it ameliorated LPS-induced sepsis in C57BL/6J mice and reduced multi-organ toxicity. Taken together, **PIF_9** was identified as a potential sepsis solution, targeting inflammation and oxidative stress through modulating MAPKs/NF-κB.

## 1. Introduction

Sepsis is caused by a series of inflammatory reactions due to the flow of pathogenic bacteria into various tissues through the bloodstream, which eventually leads to the failure of different organs [1,2]. Millions of people worldwide suffer from sepsis each year [3], but there is no effective specific treatment for it [4,5]. Owing to the essence of SIRS in sepsis, evaluating the degree of inflammatory reaction of patients can contribute to an accurate assessment of the disease stage [6]. Moreover, inflammatory cytokines, such as TNF-α and IL-1β, are first responders which can dysregulate the immune response and consequently cause damage to multiple tissues in sepsis [7,8], because they can be massively produced and released by circulating immune cells through both transcriptional and posttranslational regulation [9]. Thus, inflammatory response is a promising target in sepsis.

During the progression of sepsis, a complex array of systems are subject to secondary stimulation, including activation of the complement system, platelet-activating factors, arachidonic acid metabolites, ROS, and NO. A wealth of studies described oxidative stress in sepsis patients with the evidence of redundant ROS production and anti-oxidative depletion [10]. Under normal conditions, oxidative stress activates a series of transcription factors, which in turn induce the expression of various genes, including pro-inflammatory cytokines [11]. Therefore, as signal molecules, ROS are also mediators of inflammation [12,13], and their production is vital to the progress of many inflammatory diseases [14]. As oxygen free radicals and other ROS appear to be involved as messengers in cellular signal transduction and gene activation, then logically there will be implications for the expression and control of the immuno-inflammatory response during sepsis [15]. Moreover, oxidative stress initiates inflammatory responses by activating redox pathways; for example, increased activation of NF-κB and increased circulating inflammatory mediators including cytokines and pentraxin-3 have been identified in patients with sepsis [16,17,18]. In summary, oxidative stress should also be carefully tuned in sepsis treatment.

**Figure 1:**
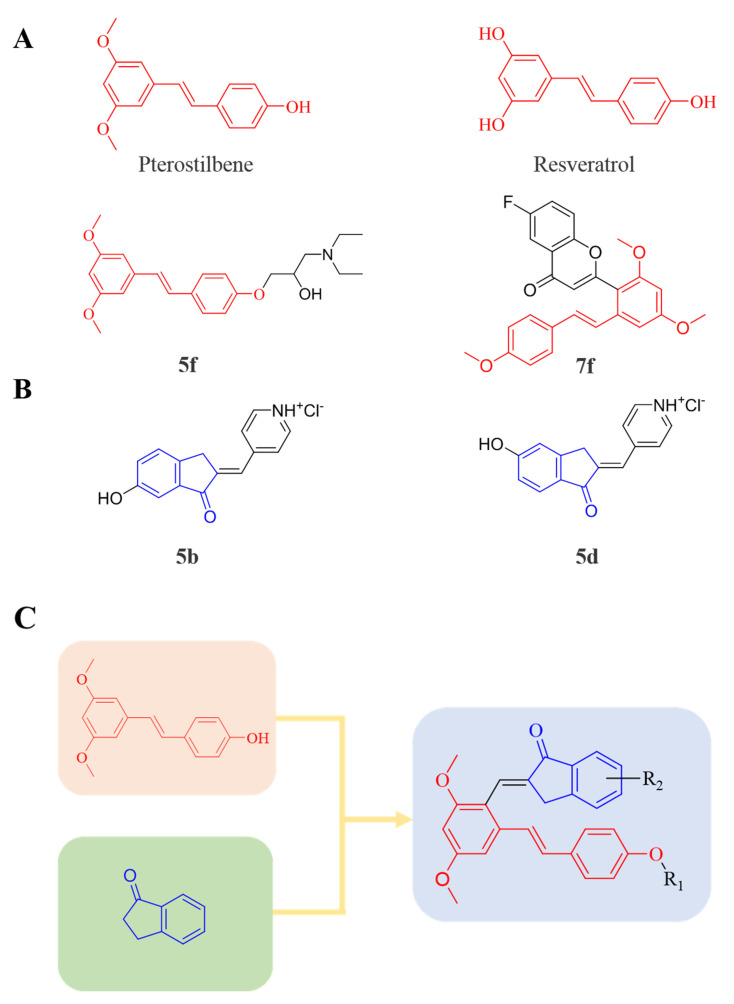
(**A**) Structures of pterostilbene, resveratrol, and their active derivatives **5f** and **7f**, respectively. (**B**) Structures of indanone active derivatives **5b** and **5d**. (**C**) Design strategy of pterostilbene indanone derivatives (the red part of the structure is pterostilbene, and the blue part is indanone).

Therefore, it might be a good strategy to discover novel antiseptics by investigating compounds that can attenuate oxidative stress and inflammatory responses. In recent years, a variety of natural products have been demonstrated to have anti-oxidative and anti-inflammatory effects. Pterostilbene (Figure 1A) is a homologue of resveratrol (Figure 1A), a classic natural polyphenolic substance, known for its attractive anti-inflammatory [19], anti-tumor, and anti-oxidative stress abilities [20]. Pterostilbene is a demethylated resveratrol derivative with better lipophilicity, stability, intestinal permeability, anti-injury ability, and bioavailability [20].

Therefore, pterostilbene can be used in the design and development of new drugs for the treatment and prevention of related diseases, and its broad application prospects are of great interest to researchers [21,22]. Zheng et al. designed and synthesized a series of pterostilbene derivatives, of which compound **5f** (Figure 1A) exhibited high anti-oxidative activity and neuroprotective effects [23]. Chen et al. designed and synthesized 37 new resveratrol flavanol derivatives, of which compound **7f** (Figure 1A) could inhibit the transduction of NF-κB cell signaling pathway caused by TLR4 activation and attenuate lipopolysaccharide-induced acute lung injury (ALI) in mice [22]. Indanones are also pharmacophores often embedded in more complex molecules with broad biological activities, including small molecules with anti-oxidative, antibacterial, and anti-inflammatory activities [24]. Man Kadayat et al. designed and synthesized a series of indanone derivatives linked to pyridine, of which compounds **5b** and **5d** (Figure 1B) exhibited the most potent inhibitory activity against TNF-α-induced monocyte adhesion to colonic epithelial cells. In a rat model of colitis, oral administration of compounds **5b** and **5d** ameliorated colitis, suggesting that **5b** and **5d** may be developed as promising drugs for the treatment of inflammatory bowel disease [25]. Both pterostilbene and indanones have proved their appealing biological characteristics separately. Based on that, we reasoned the combination of both might exert more interesting and potent functions.

Herein, in order to discover a novel series of pterostilbene derivatives with optimal anti-oxidative and anti-inflammatory activities for sepsis treatment, we introduced indanone moiety onto the pterostilbene skeleton, which is based on the strategy of pharmacophore combination (Figure 1C). Then, we used in vitro and in vivo sepsis models to evaluate the anti-oxidative and anti-inflammatory activity of the compound with the best profile in order to identify its potential mechanism of action by exploring relevant signaling pathways.

## 2. Materials and Methods

### 2.1. Chemistry

#### 2.1.1. General

In general, all reagents used in the synthesis were obtained from Aladdin, Tansoole et al. without further purifications. Reactions were monitored by analytical thin-layer chromatography (TLC) and visualized under UV light (λ = 254 or 365 nm). Purification by chromatography column was carried out using silica gel (200–300 meshes).

#### 2.1.2. General Procedure for the Preparation of **B1**–**B8**

To a stirred solution of A (10 mmol, 1.0 equiv) in acetonitrile (20 mL), anhydrous K_2_CO_3_ (10 mmol, 1.0 equiv) and corresponding R1-Br (15 mmol, 1.5 equiv) were added. The resulting reaction mixture was refluxed overnight. After completion, the reaction mixture was cooled to room temperature and diluted with water. The aqueous layer was extracted with EtOAc, washed with water and brine, dried over anhydrous Na_2_SO_4_**,** and concentrated to afford the crudes. **B1**–**B8** were then purified by column chromatography (gradient elution of PE/EtOAc = 3/1, *v*/*v*), respectively.

#### 2.1.3. General Procedure for the Preparation of **C1**–**C8**

To a stirred solution of **B** (5 mmol, 1.0 equiv) in DMF (20 mL), POCl_3_ (10 mmol, 2 equiv) was added dropwise at 0 °C for 0.5 h. Then, the reaction mixture was stirred at room temperature for 1.5 h. The reaction was monitored by TLC, and quenched immediately by ice water after completion. Then, sodium hydroxide solution was added under stirring to adjust pH to alkaline. After stirring overnight, the light-yellow solid was precipitated, filtered, and dried. **C1**–**C8** were purified by column chromatography (gradient elution of DCM/PE = 3/1, *v*/*v*), respectively.

#### 2.1.4. Synthesis of Pterostilbene-Based Indanone Derivatives **PIF_1**–**PIF_16**

To a solution of **C** (1 mmol, 1.0 equiv) and the corresponding indanones (1 mmol, 1.0 equiv) in ethanol (15 mL), 5 mL 40% KOH was added to dropwise and the reaction mixture was stirred at room temperature for 12 h. Then, the resulting mixture was slowly added to a solution of ice-water, stirred for 2 h, and filtered to obtain the crude product, which was purified by column chromatography (gradient elution of PE/EtOAc = 2/1, v/v), to afford the corresponding pure products **PIF_1**–**PIF_16**.

Their structures were characterized by ^1^H NMR, ^13^C NMR, and HR-MS. All of the synthetic compounds gave satisfactory analytical and spectroscopic data, which demonstrated in Supporting Information.

### 2.2. Reagents and Cell Culture

Lipopolysaccharides (LPS) were purchased from Sigma Aldrich (St. Louis, MO, USA). Dexamethasone (DEX), indomethacin, and BAY11-7082 were purchased from MedChemExpress (Monmouth Junction, NJ, USA). Nitric oxide (NO) assay kit was purchased from Beyotime biotechnology (Shanghai, China). Mouse anti-GAPDH, rabbit anti-iNOS, COX-2, NF-κB, p38, p-p38, JNK, p-JNK, SOD1, and β-actin antibodies were purchased from Abclonal (Wuhan, China). Rabbit anti-ERK and p-ERK antibodies were purchased from Affinity Biosciences (Cincinnati, OH, USA). RAW264.7 cells were purchased from the ATCC (Manassas, VA, USA) and cultured in complete RPMI 1640 medium containing 10% FBS and 50 μg/mL penicillin/streptomycin in a humidified incubator with 5% CO_2_ at 37 °C. The cells were switched to serum-free medium at ~90% confluence for 2 h, followed by treatment. Treatments were carried out in 96-well plates by pre-incubating the cells for 1 h with test compounds (10 μM) previously solubilized using dimethyl sulfoxide (DMSO). DMSO was used as the vehicle control in all the experiments.

### 2.3. Determination of Cell Viability and Medium NO Levels

Cell viability was detected using MTT assay [26]. After washing with PBS 3 times and incubating in DMSO (150 mL/well) for 15 min, the optical density at a wavelength of 550 nm was read by a microplate reader (BioTek Instruments, Winooski, VT, USA). Culture medium was collected for NO determination according to the manufacturer’s instructions.

### 2.4. Determination Protein and mRNA Expression

After treatment, cells were washed with PBS and lysed in lysis buffer (20 mM Tris, pH 7.5, 137 mM NaCl, 2 mM EDTA, 1% Triton X-100, 25 mM -glycerophosphate, 2 mM sodium pyrophosphate, 1 mM phenylmethylsulfonyl fluoride, 10 g/mL aprotinin/leupeptin, 100 mM NaVO4). Total RNA was extracted and stored at −80 °C; IL-1β and TNF-α mRNA levels were analyzed by qRT-PCR. Similarly, after extraction, total cellular proteins were stored at −20 °C and used to determine iNOS, COX-2, p38, p-p38, JNK, p-JNK, ERK, p-ERK, and SOD1 protein expression by Western blotting as described previously [26]. To analyze NF-κB translocation, RAW264.7 cells were cultured on coverslips in a 48-well plate. After treatment, cells were conducted by immunofluorescent staining [27].

Cellular total RNA was extracted using TRizol regent, cDNA was synthesized using the reverse transcription kit, and qRT-PCR was performed using the SYBR green PCR master mix with primers listed in Table S1 on LightCycler96 (Roche, Mannheim, Germany). Expression of mRNA was normalized by GAPDH mRNA in the corresponding sample [28,29].

### 2.5. Determination of ROS Levels in RAW264.7 Cells

After treatment, RAW264.7 cells in a 96-well plate were incubated with DCFH-DA solution (10 mM) for 20 min in the dark, then washed three times with PBS, followed by the determination of fluorescence at wavelengths of 488 nm (excitation) and 525 nm (emission) with a fluorescence microplate reader (BioTek Instruments, Winooski, VT, USA).

### 2.6. In Vivo Study

C57BL/6J mice (male, ~8-week-old) were purchased from the GemPharmatech Co., Ltd. (Nanjing, China). The protocol for animal study was approved by the Ethics Committee of Hefei University of Technology and conformed to the Guide for the Care and Use of Laboratory Animals published by NIH. All the animals were free to access food and drinking water.

C57BL/6J mice were randomly divided into five groups (6 mice/group), and received the following treatment: Groups 1 and 2 (Ctrl and LPS group), intragastric (i.g.) administration of PBS (200 μL/day) for 3 days; Group 3 (DEX group), i.g. administration of DEX solution (7 mg/day/kg bodyweight) for 3 days; Groups 4 and 5 (**PIF_9** groups), i.g. administration of **PIF_9** solution at a dose of 7 or 14 mg/day/kg bodyweight for 3 days. Mice in Group 1 were intraperitoneally (i.p.) injected with 100 μL saline, and Groups 2–5 were i.p. injected with 100 μL LPS solution (5 mg/kg bodyweight) on day 3. All mice were sacrificed 12 h after LPS injection.

### 2.7. Determination of ROS Levels in the Liver

Liver sections were subjected to DHE staining to determine the ROS levels as described and photographed with a fluorescence microscope (Leica, Wetzlar, Hesse-Darmstadt, German) [26].

### 2.8. TUNEL Assay

We conducted TUNEL assay to determine the apoptotic cells of liver tissue, and liver paraffin sections were obtained according to the instructions of the TUNEL BrightRed Apoptosis Detection kit (Vazyme Biotech Co., Ltd., Nanjing, China.). Images were acquired on an optical microscope at 20 magnification. TUNEL-positive cells were counted with five random fields per section by Image J V1.8.0 software (National Institute of Health).

### 2.9. Determination of Serum Biochemical Indicators

After collection, blood samples were kept at room temperature for 2 h, then centrifuged for 20 min at 2000 *rpm*. Serum was transferred into a new test tube and determined aspartate aminotransferase (AST), creatinine (CREA), urea nitrogen (UREA), lactate dehydrogenase (LDH), and hydroxybutyrate dehydrogenase (HBDH) levels using automatic biochemical analyzer (Hitachi, Tokyo, Japan) [27].

### 2.10. Hematoxylin and Eosin (HE) Staining

Liver, kidney, spleen, and cardiac tissues were fixed with 10% formalin, dehydrated, and embedded in paraffin for preparation of 5-μm section. Tissue sections were conducted using HE staining, the slides were observed under a Zeiss microscope, and the images were photographed [27].

### 2.11. Data and Statistical Analysis

The data and statistical analysis comply with the recommendations on experimental design and analysis in pharmacology (Curtis et al., 2015). Data are presented as mean ± SEM except where indicated, and were generated from at least five independent experiments. The density of each captured image was quantified by a technician (blinded to the treatments) with segmentation colour-threshold analysis using morphometry software (IP Lab, Scanalytics, Rockville, MD, USA) (Stein et al., 2010). For Western blot assays, the target band was normalized to GAPDH or to Lamin A/C in the corresponding sample to reduce variance. All values (control and test) were normalized to the mean value of the experimental control group. The data were expressed as folds of the control group’s mean value. The data in normal distribution, which was determined by the 1-sample K-S of the non-parametric test with SPSS 22 software, were analyzed by parametric statistics (one-way ANOVA for more than two groups). For ANOVA, Bonferroni’s post hoc test was performed for data with F at *p* < 0.05 and no significant variance inhomogeneity. Differences between group means were considered to be significant when *p* < 0.05.

## 3. Results and Discussion

### 3.1. Chemistry

**Figure 2:**
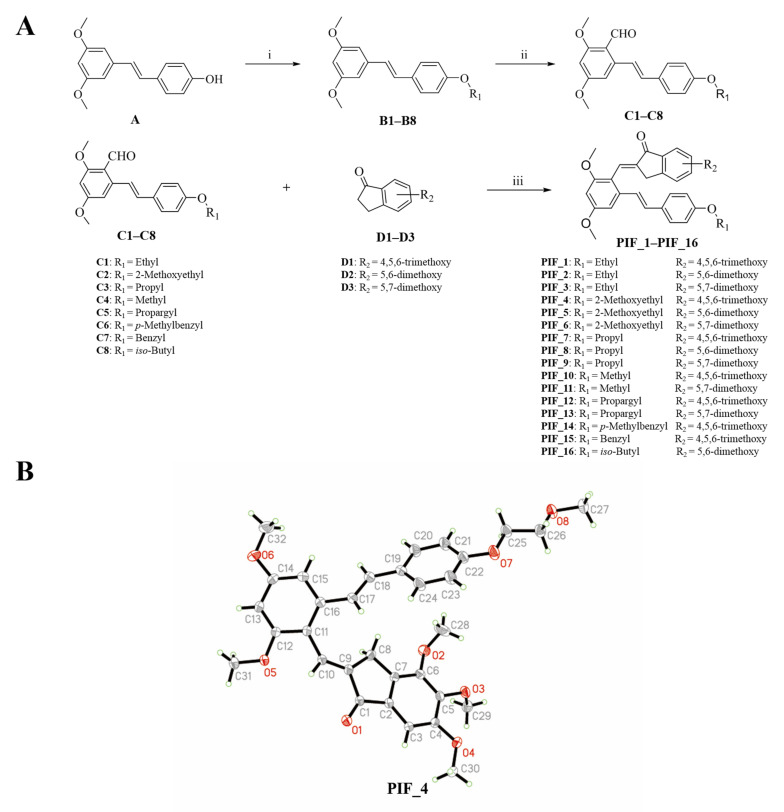
Synthetic pathway for the formation of pterostilbene indanone derivatives. (**A**) Synthetic route of the title compounds. Reagents and conditions: (i) K_2_CO_3_, Acetonitrile, reflux, 16 h. (ii) DMF, POCl_3_, neat condition, 16 h stirring at 80 °C. (iii) Ethanol, 40% KOH, neat condition, 12 h stirring at room temperature. (**B**) Schematic representation of ORTEP crystal structure view of compound **PIF_4**.

The synthetic route of pterostilbene-indanone derivatives (**PIF_1**–**PIF_16**) is shown in Figure 2A. The title compounds were prepared by the following steps: compounds (**B1**–**B8**) were obtained by reacting pterostilbene with different brominated alkanes in the presence of potassium carbonate (K_2_CO_3_) in acetonitrile under reflux conditions. The Vilsmeier reaction of compounds **B1**–**B8** used POCl_3_ and DMF afforded the key aldehyde intermediates **C1**–**C8**. Then, the reaction of resveratrol aldehyde derivatives (**C1**–**C8**) with indanone derivatives in ethanol in the presence of 40% KOH solution resulted in pterostilbene indanone derivatives **PIF_1**–**PIF_16**. All the target compounds gave satisfactory analytical and spectroscopic data, which is demonstrated in Supplementary Materials.

Moreover, the structure of compound **PIF_4** was determined by X-Ray diffraction crystallography (Figure 2B). ^1^H NMR spectrum of alkene (CH=CH, compound **PIF_4**) exhibited a chemical shift at 7.04 ppm in CDCl_3_ with a coupling constant of 16.7 Hz. These results demonstrated that the configuration of compound **PIF_4** is *E* geometrical isomer, and the result was further confirmed by crystallographic data. Crystallographic data (excluding structure factors) for the structure had been deposited with the Cambridge Crystallographic Data Center as supplementary publication (CCDC NO. 2093128). Crystal data were shown as below: yellow crystals, yield = 44.8%; m.p. 149–151 °C; C_32_H_34_O_8_, monoclinic, space group P21/n; a = 17.6968, b = 7.86505, c = 19.9830 (Å); α = 90, β = 90.6603, γ = 90 (°); V = 2781.18 Å^3^; Z = 4; F(000) = 1160.0; reflections collected/unique = 12585/4900; data/restraints/parameters = 4900/0/368; goodness of fit on F^2^ = 1.074; final R indexes, R_1_ = 0.0403, wR_2_ = 0.1190.

### 3.2. Intrinsic Cytotoxicity of the Title Compounds against RAW264.7 Cells

**Figure 3:**
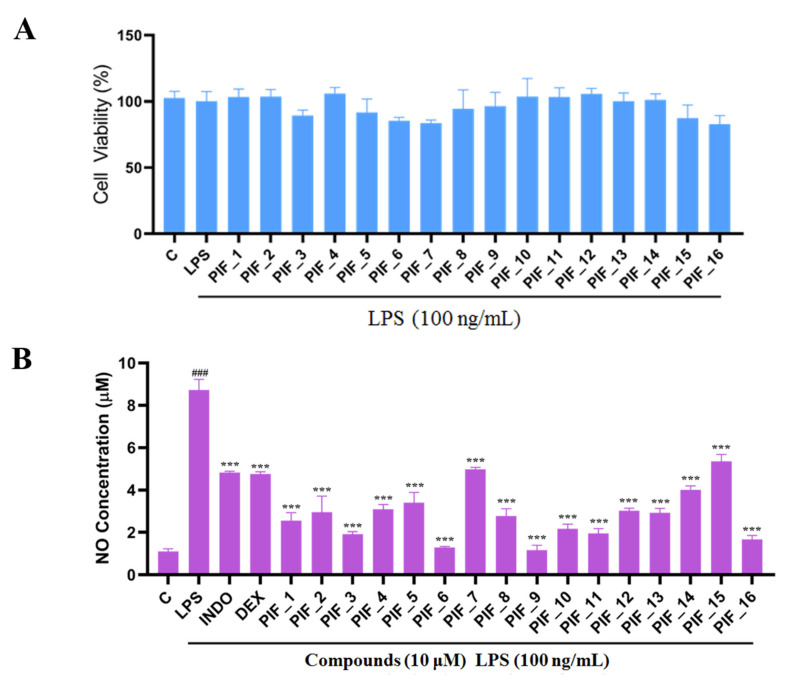
The ability of compounds to inhibit LPS-induced NO production in RAW264.7 macrophages. (**A**) The cytotoxic evaluation in RAW264.7 of compounds. RAW264.7 cells in a 96-plate cultured with serum-free medium were pre-incubated with all compounds (10 µM) for 1 h, then washed with PBS followed by MTT determination as described in Materials and Methods. (**B**) Inhibition NO production by compounds with low cytotoxicity. RAW264.7 cells in a 96-plate cultured with serum-free medium were pretreated with compounds (10 µM) for 1 h and co-treated with LPS (100 ng/mL) for 24 h. Cellular medium were collected for NO determination as described in Materials and Methods. INDO: positive control Indomethacin. DEX: positive control Dexamethasone. ^###^, *p* < 0.001 vs. Control; ***, *p* < 0.001 vs. LPS-treated group.

RAW 264.7 cells are usually used as a macrophage model in screening anti-oxidative and anti-inflammatory drugs. We performed a MTT assay to rule out any potential cytotoxic effect of **PIF_1–PIF_16** onto RAW264.7 macrophages. To this aim, cells were preincubated with the test compounds, then cultured with or without LPS for 24 h. As shown in Figure 3A, all compounds showed no significant intrinsic cytotoxicity at 100 nM, 1 µM, and 10 µM, which indicated that such concentrations could be suitable for subsequent experiments.

### 3.3. Inhibitory SAR of ***PIF_1***–***PIF_16*** against LPS-Induced NO Release

LPS is a common immune stimulant for RAW264.7, inducing the secretion of NO, and inflammatory factors such as IL-1β and TNF-α. Overreaction to the presence of LPS can lead to sepsis, septic shock, or SIRS. Excessive production of nitrogen oxides, and the formation of endogenous hydrogen peroxide from nitrogen oxides and superoxide, is thought to be a key factor in cardiac depression in sepsis [30]. It also plays an important role in many inflammatory diseases [31]. Concomitantly released NO may thus react with O_2_^•−^, yielding an even more potent oxidant, namely peroxynitrite (ONOO^•^), which in turn can be decomposed to highly reactive HO^•^ [32]. Therefore, the level of anti-inflammatory activity of compounds is usually evaluated according to the release of NO as a standard and it is also related to the level of antioxidants [33,34].

Here, in order to evaluate the inhibitory abilities of title compounds against NO production, RAW264.7 cells were pre-incubated with all compounds (10 µM). Then, Griess reagent was used to determine the concentration of NO.

We found that all the tested compounds except **PIF_7** and **PIF_15** exhibited stronger inhibition of NO production compared with the positive controls dexamethasone and indomethacin. Among them, compounds **PIF_3** (R_1_ is ethyl, R_2_ is 5,7-dimethoxy), **PIF_6** (R_1_ is 2-methoxyethyl, R_2_ is 5,7-dimethoxy), **PIF_9** (R_1_ is propyl, R_2_ is 5,7-dimethoxy), and **PIF_16** (R_1_ is *iso*-butyl, R_2_ is 5,6-dimethoxy) showed more than 2-fold inhibitory activity over the positive control. Compounds **PIF_1** (R_1_ is ethyl, R_2_ is 4,5,6-trimethoxy), **PIF_2** (R_1_ is ethyl, R_2_ is 5,6-dimethoxy), **PIF_4** (R_1_ is 2-methoxyethyl, R_2_ is 4,5,6-trimethoxy), **PIF_5 (**R_1_ is 2-methoxyethyl, R_2_ is 5,6-dimethoxy), **PIF_8** (R_1_ is propyl, R_2_ is 5,6-dimethoxy), **PIF_10** (R_1_ is methyl, R_2_ is 4,5,6-trimethoxy), **PIF_11** (R_1_ is methyl, R_2_ is 5,7-dimethoxy), **PIF_12** (R_1_ is propargyl, R_2_ is 4,5,6-trimethoxy), **PIF_13** (R_1_ is propargyl, R_2_ is 5,7-dimethoxy), and **PIF_14** (R_1_ is *p*-Methylbenzyl, R_2_ is 4, 5, 6-trimethoxy) showed moderate inhibitory activity (Table 1). These results indicated that the inhibition effect is stronger when R_1_ is an aliphatic chain with no more than three carbons than other oxygenated, unsaturated carbon chains, or aromatic substituents. Furthermore, the inhibitory activity of 5,7-dimethoxy substituted compound is better than 4,5,6-trimethoxy substituted one, and 5,6-dimethoxy substituted has slightly lower inhibitory activity. Thus, the SAR could be summarized as the substitution of an aliphatic chain with no more than three carbons at the hydroxyl group of pterostilbene, and the introduction of 5,7-dimethyl-1-indanone at the 2-position of the A-ring of pterostilbene is favorable to inhibit the NO-releasing activity.

Therefore, these compounds are of value for further evaluation. To explore the an-tioxidant and anti-inflammatory mechanisms of the synthetic compounds in vitro and in vivo, the original compound **PIF_9** with the highest inhibitory activity was selected for the following studies based on cellular NO inhibitory activity and cytotoxicity.

### 3.4. Dose-Dependent Inhibition of NO and Cytokine Production by the Active Compound ***PIF_9***

TNF-α is a cytokine involved in systemic inflammation during the acute phase reaction. It is mainly secreted by macrophages. IL-1β, also known as catabolic enzyme, is a member of the interleukin-1 cytokine family [35]. TNF-α and IL-1β are two well-known pro-inflammatory cytokines. They bring about an inflammatory cascade response, amplify the effects, exacerbate the inflammatory response, and play an important role in the pathological development of many inflammatory diseases [34,36]. TNF-α and IL-1β can induce ROS production and aggravate oxidative stress. Since TNF-α and IL-1β are identified as keystone cytokines in the inflammatory process and oxidative stress, we further evaluated the expression of TNF-α and IL-1β expression at protein level.

**Figure 4:**
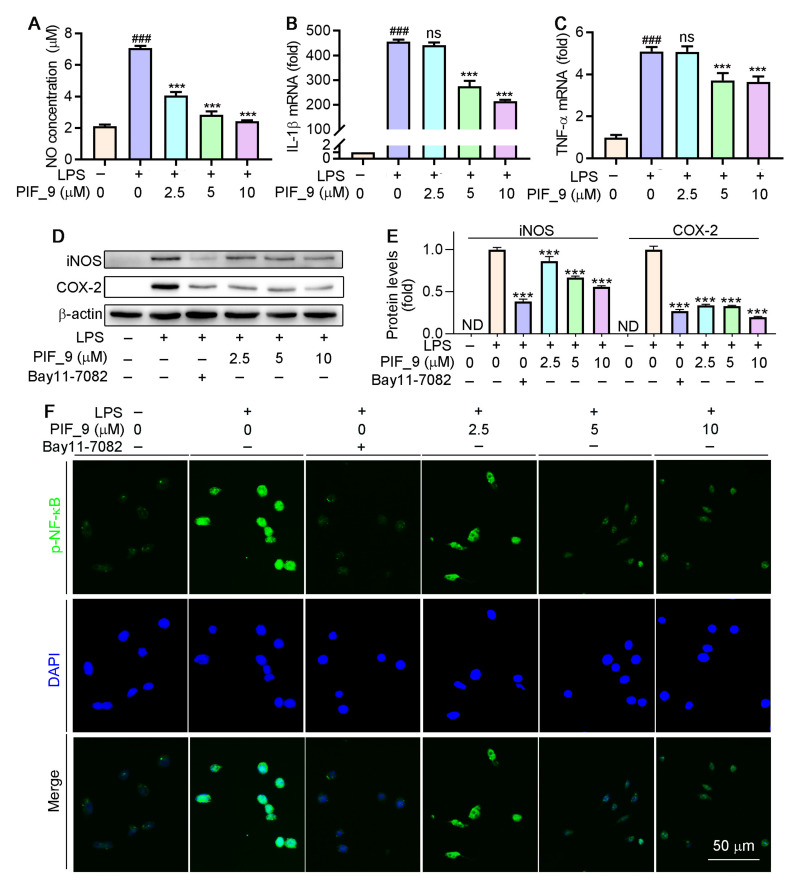
Compound **PIF_9** inhibited LPS-induced inflammation in RAW264.7 cells by reducing NF-κB activation. RAW264.7 cells in a 96-well plate (**A**) or a 6-well plate (**B**–**E**) or a 48-plate with coverslips (**F**) cultured in serum-free medium were pretreated with compound **PIF_9** for 1 h, then co-treated with LPS (100 ng/mL) for 24 h. Medium NO levels were detected by NO assay kit (**A**). After treatment, total RNA was extracted; IL-1β (**B**) and TNF-α (**C**) mRNA levels were analyzed by qRT-PCR as described in Materials and Methods. (**D**,**E**) Protein expression of iNOS and COX-2 were analyzed by Western blot followed by quantitative analysis of band density as described in Materials and Methods. (**F**) Expression of p-NF-κB was conducted by immunofluorescence staining as described in Materials and Methods. ^###^, *p* < 0.001 vs. Control; ***, *p* < 0.001 vs. LPS-treated group; ND, no detection. ns, no significant difference (*n* = 3).

Different concentrations (2.5, 5, and 10 µM) of compound **PIF_9** were selected for further study. As shown in Figure 4A–C, compound **PIF_9** inhibited NO production, and mRNA levels of IL-1β and TNF-α in LPS-stimulated RAW264.7 cells in a dose-dependent manner. Nikhil et al. [19] have shown that pterostilbene derivatives have an inhibitory effect on the release of NO and inflammatory factors, as confirmed by our experimental results. These results further proved that compound **PIF_9** was able to inhibit the expression of inflammatory mediators (TNF-α and IL-1β) in addition to NO. Combining the results of NO and cytokine inhibition, we identified **PIF_9** as the most representative compound for further mechanism exploration.

### 3.5. Assessment of the Ability of Compound ***PIF_9*** to Inhibit LPS-Induced iNOS and COX-2 Upregulation

Inducible nitric oxide synthase (iNOS) and cyclooxygenase-2 (COX-2) are key regulators of inflammatory mediator production in response to LPS and other stimuli [37]. INOS exists in a variety of cells and is highly expressed by external stimuli such as pro-inflammatory cytokines and LPS. Overexpression of iNOS will lead to excessive production of NO. Over-produced NO and O_2_^•−^ generate the powerful oxidant and nitrifier ONOOO^−^. The latter leads to nitrification of proteins and DNA damage through oxidative deamination [38]. COX-2 regulates prostaglandin synthesis and its expression is induced by cytokines such as TNF-α, IL-1β, and IL-6 [39]. We next evaluated the ability of compound **PIF_9** to modulate LPS-induced iNOS and COX-2 expression by treating RAW264.7 cells for 24 h with **PIF_9** (2.5, 5, or 10 µM) and LPS (100 ng/mL). As shown in Figure 4D,E, the levels of these two proteins were then assessed via Western blot. LPS treatment resulted in a significant increase in the protein levels of both iNOS and COX-2, whereas this phenomenon was reversed by **PIF_9** in a dose-dependent manner.

Chen et al. [22] confirmed that resveratrol derivatives, structurally similar to **PIF_9**, have been shown to inhibit LPS-induced iNOS and COX-2 expression in a concentration-dependent manner, which is in good agreement with our results. As we expected, the results of the present study also proved this. Therefore, our results suggest that **PIF_9** can be a promising antioxidant and anti-inflammatory agent that is capable of suppressing LPS-induced signaling activity.

### 3.6. Effect of Compound ***PIF_9*** on LPS-Induced Activation of NF-κB and MAPKs

Previous studies have demonstrated that LPS-induced ROS production is associated with the activation of NF-κB [40]. As a common well-known transcription regulator, NF-κB plays a key role in both inflammatory responses and cell differentiation, proliferation, and apoptosis [41]. In general, NF-κB is present in a latent state, in which it binds to p50-p65 heterodimer protein primarily in the form of IκB, an inhibitor of NF-κB [42]. However, a number of stimuli, such as ROS, TNF, antigens, and interleukin, can activate NF-κB [43]. Inappropriately, increased or prolonged activation of NF-κB, resulting in the overexpression of mediator proteins, may account for the deleterious effects in sepsis [44]. Here, we used immunofluorescence staining to explore the effects of compound **PIF_9** on the NF-κB pathway. As shown in Figure 4F, compared with LPS treatment alone, the enhanced green fluorescence signal in **PIF_9**-treated cells was significantly decreased. This finding suggests that **PIF_9** could inhibit nuclear translocation and activation of κB.

**Figure 5:**
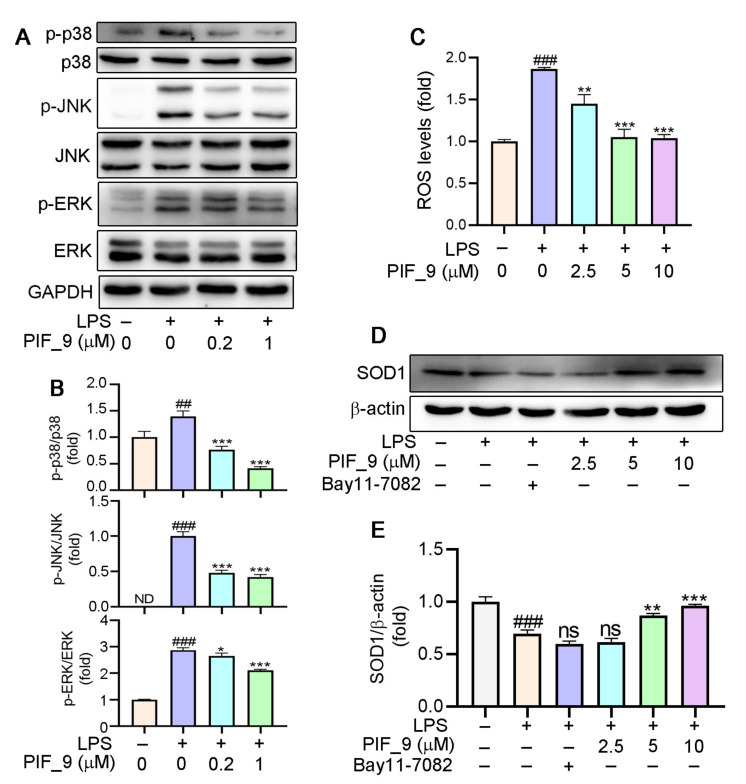
(**A**,**B**) Compound **PIF_9** inhibited the activation of MAPK signaling pathway. RAW264.7 cells in a 6-well plate cultured in serum-free medium were pretreated with compound **PIF_9** for 1 h, then co-treated with LPS (100 ng/mL) for 2 h. (**C**) RAW264.7 cells were pretreated with compound **PIF_9** for 1 h, then co-treated with LPS (100 ng/mL) for 24 h. Cellular ROS levels was determined by detection of DCF fluorescence intensity as described in Materials and Methods. (**D**,**E**) RAW264.7 cells were pretreated with compound PIF_9 for 1 h, then co-treated with LPS (100 ng/mL) for 24 h. After treatment, cellular protein was extracted and protein expression levels of p-JNK, JNK, p-p38, p38, p-ERK, ERK (A,B), and SOD1 (D, E) were analyzed by Western blot, followed by quantitative analysis of band density as described in Materials and Methods. ^##^, *p* < 0.01, ^###^, *p* < 0.001, vs. Control; *, *p* < 0.05, **, *p* < 0.01, ***, *p* < 0.001 vs. LPS-treated group; ns, no significant difference; ND, no detection (*n* = 3).

Mitogen-activated protein kinases (MAPKs) are important intracellular signal transduction members that regulate inflammatory gene expression, and that includes singling intermediaries such as JNK (c-Jun N-terminal kinase), ERK1/2 (extracellular regulated protein kinases), and p38 [45]. MAPK activation can drive the upregulation of proteins including iNOS and COX-2 [37]. Microglial activation triggered by LPS involves a number of signaling pathways, which include MAPK family. This allows the transcription factor AP-1 to translocate into the nucleus and bind to target promoters, turning on transcription of inflammatory genes, including TNF-α, IL-6, and other inflammatory mediators. Furthermore, MAPKs such as p38 and JNK are also redox-sensitive [40]. Nikhil et al. [19] have shown that pterostilbene and its derivatives have an effect on LPS-induced phosphorylation of MAPKs [19], and we have also conducted a more in-depth mechanistic exploration. Therefore, we analyzed it by Western blot. The results showed that **PIF_9** could dose-dependently block LPS-induced ERK phosphorylation, JNK phosphorylation, and p38 phosphorylation (Figure 5A,B). These results indicated that compound **PIF_9** has anti-oxidative and anti-inflammatory effects, which may be through modulating MAPKs and NF-κB signaling pathway.

### 3.7. Anti-Oxidantive Activities of the Compound ***PIF_9***

It is well-known that ROS are products of normal cell metabolism, and an excessive amount of ROS can cause oxidative stress [46]. An accumulation of ROS may destroy the balance between anti-oxidative and pro-oxidant systems, and then activate various defense mechanisms in cells [47]. Previous studies have shown that the increase of ROS is related to the development of inflammation. Mounting evidence also indicates that oxidative stress and inflammation are inextricably intertwined, with complicated feedforward and feedback loops [19,48,49]. On this basis, we investigated the effects of compound **PIF_9** on intracellular ROS levels. As expected, LPS treatment was shown to significantly increase ROS content. However, pretreatment with **PIF_9** of 2.5 µM for 1h inhibited this effect. In addition, cells treated with compound **PIF_9** at 5 and 10 µM significantly reduced ROS expression. (Figure 5C). This result is also consistent with our previous studies [31].

Superoxide dismutase 1 (SOD1) is part of an enzymatic defense system against oxidative decay and functions by turning superoxide radical anions into H_2_O_2_.Recent studies proved that SOD1 is a major anti-oxidative enzyme in controlling oxidative stress in conditions of renal injury and it can also inhibit the response of pro-inflammatory cytokines [15]. Consequently, the protein expression of SOD1 is also one of the important indexes to evaluate the anti-oxidative level of compounds [50]. Our results showed that the expression of SOD1 protein was significantly inhibited by LPS treatment, but increased significantly by **PIF_9** (Figure 5D,E). Our findings suggest that **PIF_9** can limit ROS production and upregulate SOD1 expression in an inflammatory content, which may be a promising lead for the development of novel antioxidant and anti-inflammatory agents to attenuate oxidative stress and inflammation-induced diseases.

### 3.8. Compound ***PIF_9*** Improves LPS-Induced Sepsis in C57BL/6J Mice

**Figure 6:**
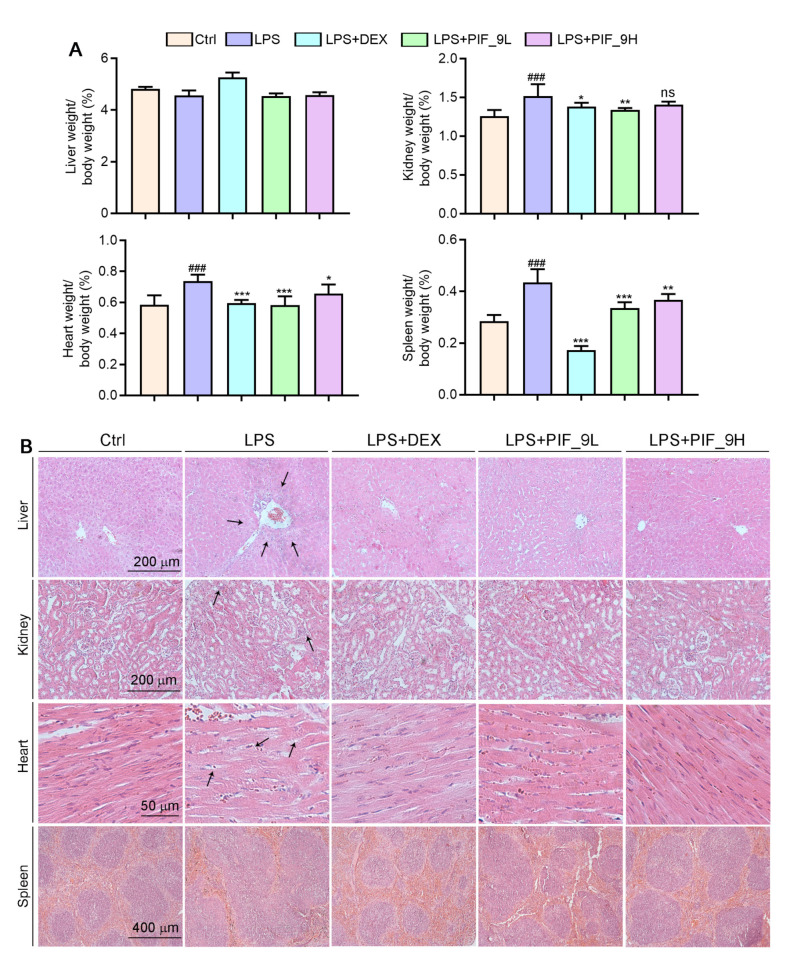
Compound **PIF_9** improved multiple tissues damage in sepsis mice. (**A**) At the end of the experiment, liver, kidney, heart, and spleen tissues were collected and weighted and the ratio of tissue weight to body weight was calculated. (**B**) Measurements of 5-μm paraffin sections of liver, kidney, heart, and spleen tissues were conducted using HE staining as described in Materials and Methods; the representative photographs are presented. The black arrows indicate tissue damage. ^###^, *p* < 0.001 vs. Control group; *, *p* < 0.05, **, *p* < 0.01, ***, *p* < 0.001 vs. LPS group (*n* = 6). ns, no significant difference.

As a major endotoxin, LPS from Ggram-negative bacteria has been implicated as a major cause of sepsis. Thus, based on in vitro anti-inflammatory and anti-oxidant effects of compound **PIF_9**, we constructed septic animal models using C57BL/6J mice and treated them with **PIF_9** to for evaluate its therapeutic effect in vivo. The results showed that the ratios of kidney, spleen, and heart weight to body weight were significantly increased in the LPS-induced group. However, compared with the LPS-induced group, low concentrations of **PIF_9** significantly reduced the kidney, spleen, and heart weight to body weight ratios. High concentrations of **PIF_9** also have a good down-regulating effect on the kidney, spleen, and heart weight to body weight ratios (Figure 6A). In addition, we performed HE staining to observe the pathomorphological changes of different tissues. As shown in Figure 6B, we found that LPS enhanced inflammatory infiltration of liver tissue and induced morphological changes in renal tubules and cardiomyocytes. These changes were attenuated by the compound **PIF_9**. In the spleen, LPS promoted the expansion and fusion of white bone marrow, blurring its borders. In contrast, treatment with the compound **PIF_9** ameliorated these changes in spleen tissue.

**Figure 7:**
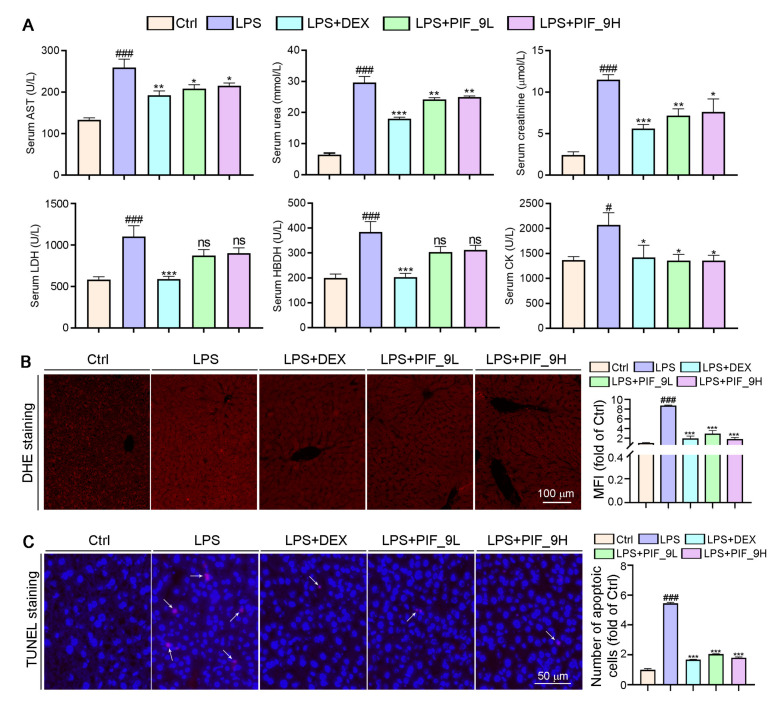
Compound **PIF_9** reduced serum Urea, Crea, LDH, HBDH, and AST levels in sepsis mice. (**A**) At the end of the experiment, the serum was collected and diluted with PBS, followed by a determination of AST, UREA, CREA, LDH, and HBDH levels using automatic biochemical analyzer. (**B**) Measurements of 5-μm liver paraffin sections were stained with DHE staining for determination of ROS levels as described in Materials and Methods; the representative photographs are presented. (**C**) TUNEL assay with quantitation of cell apoptosis in mouse liver tissues as described in Materials and Methods. The white arrows indicate apoptotic cells. The data were conducted with one-way ANOVA followed by hoc Bartlett’s test. ^#^, *p* < 0.05, ^###^, *p* < 0.001 vs. Control group; *, *p* < 0.05, **, *p* < 0.01, ***, *p* < 0.001 vs. LPS group; ns, no significant difference (*n* = 6).

AST is found mainly in the heart muscle and, to a lesser extent, in tissues such as the liver, skeletal muscle, and kidney. The AST levels in serum are low in normal conditions, but when the corresponding cells are damaged, the cell membrane permeability increases and the intracellular plasma AST is released into the blood, so its serum concentration can be increased. UREA and CREA are the two most important indicators of kidney function [51]. When kidney function is impaired, excessive UREA and CREA are excreted into the blood, so their concentration in the blood indirectly reflects the excretory function of the kidneys. In addition, in the event of myocardial infarction and myocarditis, it causes an increase in serum concentrations of LDH and HBDH. Therefore, we used a fully automated biochemical assay to detect serum biochemical indicators. Interestingly, we found that LPS induced an increase in serum levels of AST, UREA, CREA, LDH, and HBDH, while the compound **PIF_9** significantly reduced the levels of these indicators (Figure 7A). Our study shows that compound **PIF_9** protects against LPS-induced sepsis by ameliorating systemic tissue damage.

Sepsis-induced liver dysfunction is an important risk factor for multi-organ failure and sepsis-induced mortality. Reducing liver injury and restoring liver function can reduce mortality in patients with sepsis [52]. Apoptosis is the first cellular response of the liver to pathogens and ischemic injury, followed by necrosis [53]. LPS causes apoptosis and overproduction of pro-inflammatory cytokines, leading to inflammatory tissue damage. Doğanyiğit et al. [54]. showed an increase in hepatocyte apoptosis following LPS treatment, with areas of inflammation, hemorrhage, necrosis, and infiltration in liver tissue, as well as apoptotic vesicles. To determine whether **PIF_9** treatment can inhibit sepsis-induced liver dysfunction and liver tissue damage, we conducted TUNEL assay. As shown in Figure 7C, the number of apoptotic cells was significantly increased after LPS treatment alone, which is consistent with the literature. In the current study, we showed that **PIF_9** treatment obviously decreased the number of TUNEL-positive cells in liver tissue. These results suggest that **PIF_9** can ameliorate hepatocellular apoptosis.

Accumulating studies have shown that excess ROS are mainly produced in mitochondria and play a key role in a variety of diseases such as cardiovascular disease, diabetes, and obesity [55]. The production of ROS can be induced by cytokines such as TNF-α. In turn, ROS can enhance the production of pro-inflammatory cytokines and the activation of NF-κB [16]. Previous studies suggested that both alcohol and CCl_4_ can induce inflammation and liver oxidative stress, along with the induction of inflammatory cytokines and anti-oxidative enzymes [56]. Herein, we performed DHE staining on liver sections to determine ROS levels. As shown in Figure 7B, after LPS treatment alone, a large amount of ROS in tissues was stained to show red fluorescence, which was significantly attenuated in the compound **PIF_9**-treated group. Taken together, we demonstrate that **PIF_9** protects mice from LPS-induced sepsis by ameliorating systemic tissue damage and inhibiting ROS production.

## 4. Conclusions

In the present study, we introduced the indanone moiety into the skeleton of pterostilbene based on the strategy of pharmacophore combination and synthesized a series of pterostilbene indanone derivatives possessing anti-oxidative and anti-inflammatory activities for sepsis treatment. Firstly, the inhibitory activities of the title compounds against LPS-induced NO release in macrophages were screened. Among them, compound **PIF_9** exhibited the most potent inhibitory activity and the SAR study showed that the propyl group at R_1_ position and the 5, 7-dimethoxy group at R_2_ position were the optimal substituents. Secondly, compound **PIF_9** effectively inhibited LPS-induced expression of inflammation-associated factors including IL-1β, TNF-α, iNOS, and COX-2 in a dose-dependent manner. Similarly, the results of Western blot and immunofluorescence staining showed that compound **PIF_9** may exert anti-oxidative and anti-inflammatory effects by modulating MAPKs and NF-κB signaling pathways. In addition, **PIF_9** could reduce cellular ROS levels and increase SOD1 expression. Finally, **PIF_9** reduced kidney, spleen, and heart weight to body weight ratios, serum biochemical parameters, liver apoptosis, and ROS levels in C57BL/6J mice. These results suggest that compound **PIF_9** ameliorates systemic tissue damage in septic mice and has a protective effect against LPS-induced sepsis. In conclusion, the compound **PIF_9** has the potential to be developed as an anti-inflammatory agent and antioxidant for the treatment of sepsis.

## Figures and Tables

**Table 1:** Chemical structures of compounds **PIF_1–PIF_16**.

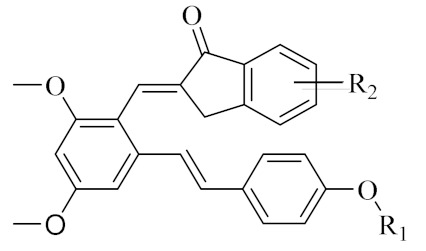 PIF_1–PIF_16					
Compd.	R_1_	R_2_	Compd.	R_1_	R_2_
**PIF_1**	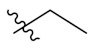	**4,5,6-OCH_3_**	**PIF_9**	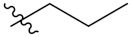	**5,7-OCH_3_**
**PIF_2**	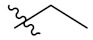	**5,6-OCH_3_**	**PIF_10**	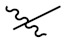	**4,5,6-OCH_3_**
**PIF_3**	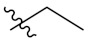	**5,7-OCH_3_**	**PIF_11**	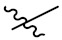	**5,7-OCH_3_**
**PIF_4**	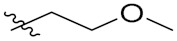	**4,5,6-OCH_3_**	**PIF_12**	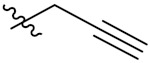	**4,5,6-OCH_3_**
**PIF_5**	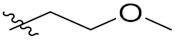	**5,6-OCH_3_**	**PIF_13**	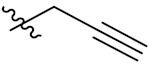	**5,7-OCH_3_**
**PIF_6**	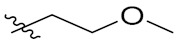	**5,7-OCH_3_**	**PIF_14**	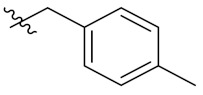	**4,5,6-OCH_3_**
**PIF_7**	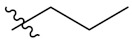	**4,5,6-OCH_3_**	**PIF_15**	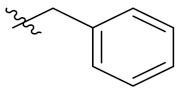	**4,5,6-OCH_3_**
**PIF_8**	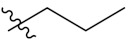	**5,6-OCH_3_**	**PIF_16**	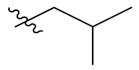	**5,6-OCH3**

## Data Availability

Supplementary Materials is attached in this submission, including the sequences of primers for qRT-PCR analysis; ^1^H-NMR, ^13^C-NMR, and HR-MS data and spectra of title compounds. CCDC NO. 2093128 contains the supplementary crystallographic data for compound **PIF_4** in this paper. The data can be obtained free of charge from the CCDC (URL: http://www.ccdc.cam.ac.uk/conts/retrieving.html (accessed on 29 June 2021).

## Supplementary Materials

The following are available online at https://www.mdpi.com/article/10.3390/antiox10091333/s1, Table S1: The sequences of primers for qRT-PCR analysis, The second part: ^1^H NMR, ^13^C NMR and HR-MS data, The third part: ^1^H NMR, ^13^C NMR and HR-MS spectra of representative compounds.
