# Supplementary material for: Discovery of Novel Pterostilbene Derivatives That Might Treat Sepsis by Attenuating Oxidative Stress and Inflammation through Modulation of MAPKs/NF-κB Signaling Pathways
Source: Antioxidants (Basel). 2021 Aug 24;10(9):1333. doi: 10.3390/antiox10091333 (PMC8470242; doi:10.3390/antiox10091333)

Supplementary Materials

Discovery of Novel Pterostilbene Derivatives That Might Treat Sepsis by Attenuating Oxidative Stress and Inflammation through Modulation of MAPKs/NF-κB Signaling Pathways

Mengyuan Fang[1, #], Tingfeng Zou[1, #], Xiaoxiao Yang[1, *], Zhen Zhang[1], Peichang Cao[1], Jihong Han[1], Yajun Duan[1], Ban-Feng Ruan[2, *], Qing-Shan Li[1, *]

[1] *Key Laboratory of Metabolism and Regulation for Major Diseases of Anhui Higher Education Institutes, College of Food and Biological Engineering, Hefei University of Technology, Hefei, 230601, P.R. China*

[2] *Key Lab of Biofabrication of Anhui Higher Education Institution Centre for Advanced Biofabrication, Hefei University, Hefei, 230601, P.R. China*

[#] *Both authors Mengyuan Fang and Tingfeng Zou contributed equally to this work.*

[*]*Corresponding authors: Qing-Shan Li, e-mail: liqs@hfut.edu.cn; Ban-Feng Ruan, e-mail: ruanbf@hfuu.edu.cn; Xiaoxiao Yang, e-mail: yangxiaoxiao@hfut.edu.cn;*

Contents

Table S1. The sequences of primers for qRT-PCR analysis. 3

^{1}H NMR, ^{13}C NMR and HR-MS data. 4

^{1}H NMR, ^{13}C NMR and HR-MS spectra of representative compounds. 11

1. Table S1. The sequences of primers for qRT-PCR analysis.

Gene	Forward primer	Reverse primer
m-IL-1β	GACCTTCCAGGATGAGGACA	AGCTCATATGGGTCCGACAG
m-TNF-α	CGTCGTAGCAAACCACCAAG	TTGAAGAGAACCTGGGAGTAGACA
m-GAPDH	ACCCAGAAGACTGTGGATGG	ACACATTGGGGGTAGGAACA

2. ^{1}H NMR, ^{13}C NMR and HR-MS data.

^{1}H and ^{13}C NMR (nuclear magnetic resonance) spectra were recorded using an Agilent 400 MHz spectrometer (Agilent Technologies, Palo Alto, CA) with $CDCl_3$ or DMSO-d_6 as the solvents and TMS as the internal standard. The signals were quoted as follows: chemical shift, multiplicity (s = singlet, d = doublet, t = triplet, q = quartet, dd = double of doublets, m = multiplet), coupling constants, and integration. High-resolution electron impact mass spectra (HR-MS) were recorded under electron impact (70 eV) condition by Micro Mass GCT CA 055 instrument. Melting points were determined on a XT4MP apparatus (Taike Corp., Beijing, China), and uncorrected.

2-((E)-2-((E)-4-ethoxystyryl)-4,6-dimethoxybenzylidene)-4,5,6-trimethoxy-2,3-dihydro-1H-inden-1-one (**PIF_1**). Yellow solid, Yield: 61.2%, m.p. 165-167°C. ^{1}H NMR (400 MHz, $CDCl_3$) δ (ppm) 7.82 (t, J = 2.0 Hz, 1H), 7.41 – 7.35 (m, 2H), 7.28 (s, 1H), 7.21 (s, 1H), 7.10 – 6.97 (m, 2H), 6.86 – 6.81 (m, 3H), 6.46 (d, J = 2.2 Hz, 1H), 4.03 (q, J = 7.0 Hz, 2H), 3.94 (dd, J = 5.5, 3.0 Hz, 9H), 3.84 (d, J = 5.4 Hz, 3H), 3.78 (s, 3H), 3.48 (d, J = 2.0 Hz, 2H), 1.42 (t, J = 7.0 Hz, 3H).^{13}C NMR (101 MHz, $CDCl_3$) δ (ppm) 192.75, 161.02, 158.95, 158.89, 154.09, 149.74, 147.56, 139.03, 138.70, 136.45, 133.96, 130.59, 129.76, 129.68, 127.97, 124.90, 116.86, 114.68, 101.59, 101.44, 97.51, 63.48, 61.13, 60.46, 56.31, 55.66, 55.48, 28.67, 14.80. HR-MS (ESI): calcd. for $C_{31}H_{32}O_7$, $[M + H]^+$, 517.2221; found 517.2282.

2-((E)-2-((E)-4-ethoxystyryl)-4,6-dimethoxybenzylidene)-5,6-dimethoxy-2,3-dihydro-1H-inden-1-one (**PIF_2**). Yellow solid, Yield: 55.7%, m.p.: 161-162°C. ^{1}H NMR (400 MHz, $CDCl_3$) δ (ppm) 7.79 (t, J = 1.8 Hz, 1H), 7.41 – 7.35 (m, 3H), 7.11 – 7.00 (m, 2H), 6.85 (dd, J = 5.5, 3.2 Hz, 3H), 6.80 (s, 1H), 6.45 (d, J = 2.1 Hz, 1H), 4.04 (q, J = 7.0 Hz, 2H), 3.96 (s, 3H), 3.93 (s, 6H), 3.85 (s, 3H), 3.48 (d, J = 1.4 Hz, 2H), 1.42 (t, J = 7.0 Hz, 3H).^{13}C NMR (101 MHz, $CDCl_3$) δ (ppm) 192.59, 160.89, 158.96, 158.94, 155.20, 149.35, 145.39, 139.72, 138.50, 131.45, 130.23, 129.69, 128.61, 127.97, 124.83, 117.18, 114.72, 107.26, 105.05, 101.32, 97.49, 63.49, 56.20, 56.17, 55.64,

55.46, 31.52, 14.80. MS (ESI): calcd. for $C_{30}H_{30}O_6$, $[M + H]^+$, 487.2115; found 487.2111

2-((E)-2-((E)-4-ethoxystyryl)-4,6-dimethoxybenzylidene)-5,7-dimethoxy-2,3-dihydro-1H-inden-1-one (**PIF_3**). Yellow solid, Yield: 50.3%, m.p.: 177-179°C. ^1H NMR (400 MHz, $CDCl_3$) δ (ppm) 7.71 (t, *J* = 2.0 Hz, 1H), 7.42 – 7.36 (m, 2H), 7.05 (s, 2H), 6.85 (dd, *J* = 9.2, 2.4 Hz, 3H), 6.44 (d, *J* = 2.2 Hz, 1H), 6.40 (d, *J* = 1.6 Hz, 1H), 6.33 (d, *J* = 1.7 Hz, 1H), 4.04 (q, *J* = 7.0 Hz, 2H), 3.97 (s, 3H), 3.92 (s, 3H), 3.84 (d, *J* = 7.2 Hz, 6H), 3.47 (d, *J* = 1.7 Hz, 2H), 1.42 (t, *J* = 7.0 Hz, 3H). ^{13}C NMR (101 MHz, $CDCl_3$) δ (ppm) 190.31, 166.64, 160.72, 160.08, 158.91, 158.78, 154.99, 139.77, 138.51, 130.09, 129.71, 127.97, 127.48, 124.74, 121.01, 117.26, 114.69, 101.43, 101.16, 97.52, 97.34, 63.48, 55.84, 55.70, 55.63, 55.45, 32.12, 14.82. MS (ESI): calcd. for $C_{30}H_{30}O_6$, $[M + H]^+$, 487.2115; found 487.2166

2-((E)-2,4-dimethoxy-6-((E)-4-(2-methoxyethoxy)styryl)benzylidene)-4,5,6-trimethoxy-2,3-dihydro-1H-inden-1-one (**PIF_4**). Yellow solid, Yield: 44.8%, m.p.: 149-151°C. ^1H NMR (400 MHz, $CDCl_3$) δ (ppm) 7.81 (s, 1H), 7.38 (d, J = 8.8 Hz, 2H), 7.21 (s, 1H), 7.04 (q, J = 16.2 Hz, 2H), 6.88 (d, J = 8.8 Hz, 2H), 6.84 (d, J = 2.2 Hz, 1H), 6.46 (d, J = 2.2 Hz, 1H), 4.15 – 4.10 (m, 2H), 3.96 – 3.92 (m, 9H), 3.85 (s, 3H), 3.79 – 3.74 (m, 5H), 3.48 (d, J = 2.0 Hz, 2H), 3.46 (s, 3H). ^{13}C NMR (101 MHz, $CDCl_3$) δ (ppm) 192.74, 161.02, 158.88, 158.75, 154.10, 149.74, 147.57, 139.04, 138.65, 136.44, 133.95, 130.50, 130.04, 129.73, 127.94, 125.09, 116.88, 114.82, 101.59, 101.46, 97.55, 70.97, 67.27, 61.14, 60.47, 59.27, 56.31, 55.66, 55.48, 28.66. MS (ESI): calcd. for $C_{32}H_{34}O_8$, $[M + H]^+$, 547.2326; found 547.2382

2-((E)-2,4-dimethoxy-6-((E)-4-(2-methoxyethoxy)styryl)benzylidene)-5,6-dimethoxy-2,3-dihydro-1H-inden-1-one (**PIF_5**). Yellow solid, Yield: 42.3%, m.p.: 171-174°C. ^1H NMR (400 MHz, $CDCl_3$) δ (ppm) 7.78 (s, 1H), 7.43 – 7.33 (m, 3H), 7.03 (d, *J* = 16.7 Hz, 2H), 6.92 – 6.82 (m, 3H), 6.80 (s, 1H), 6.45 (s, 1H), 4.18 – 4.10 (m, 3H), 3.95

(d, *J* = 12.0 Hz, 9H), 3.84 (s, 3H), 3.79 – 3.71 (m, 2H), 3.54 – 3.40 (m, 5H). ^{13}C NMR (101 MHz, $CDCl_3$) δ (ppm) 192.59, 160.89, 158.93, 158.77, 155.21, 149.36, 145.39, 139.74, 138.45, 131.44, 130.16, 130.06, 128.58, 127.94, 125.04, 117.20, 114.86, 107.25, 105.05, 101.34, 97.53, 70.98, 67.30, 60.41, 59.25, 56.21, 56.18, 55.64, 55.47, 31.52, 21.08, 14.21. MS (ESI): calcd. for $C_{31}H_{32}O_7$, $[M + H]^+$, 517.2221; found 517.2267

2-((E)-2,4-dimethoxy-6-((E)-4-(2-methoxyethoxy)styryl)benzylidene)-5,7-dimethoxy-2,3-dihydro-1H-inden-1-one (**PIF_6**). Yellow solid, Yield: 55.7%, Mp: 155-158°C. ^{1}H NMR (400 MHz, $CDCl_3$) δ (ppm) 7.71 (d, *J* = 2.0 Hz, 1H), 7.38 (dd, *J* = 8.6, 4.2 Hz, 2H), 7.04 (d, *J* = 4.2 Hz, 2H), 6.88 (dd, *J* = 8.6, 4.2 Hz, 2H), 6.83 (d, *J* = 2.1 Hz, 1H), 6.40 (dd, *J* = 30.9, 15.4 Hz, 3H), 4.13 (dd, *J* = 9.1, 4.4 Hz, 2H), 3.96 (d, *J* = 4.3 Hz, 3H), 3.91 (d, *J* = 4.4 Hz, 3H), 3.83 (dd, *J* = 7.5, 4.3 Hz, 6H), 3.76 (dd, *J* = 9.1, 4.4 Hz, 2H), 3.46 (d, *J* = 4.4 Hz, 5H). ^{13}C NMR (101 MHz, $CDCl_3$) δ (ppm) 190.25, 166.65, 160.73, 160.09, 158.78, 158.72, 154.96, 139.78, 138.48, 130.10, 130.03, 127.93, 127.43, 124.98, 121.03, 117.31, 114.85, 101.44, 101.24, 97.59, 97.36, 70.99, 67.29, 59.24, 55.83, 55.68, 55.63, 55.44, 32.10. MS (ESI): calcd. for $C_{31}H_{32}O_7$, $[M + H]^+$, 517.2221; found 517.2285

2-((E)-2,4-dimethoxy-6-((E)-4-propoxystyryl)benzylidene)-4,5,6-trimethoxy-2,3-dihydro-1H-inden-1-one (**PIF_7**). Yellow solid, Yield: 58.9%, m.p.: 188-190°C. ^{1}H NMR (400 MHz, $CDCl_3$) δ (ppm) 7.82 (t, *J* = 2.0 Hz, 1H), 7.42 – 7.35 (m, 2H), 7.21 (s, 1H), 7.04 (q, *J* = 16.2 Hz, 2H), 6.87 – 6.81 (m, 3H), 6.46 (d, *J* = 2.2 Hz, 1H), 3.95 – 3.92 (m, 10H), 3.85 (s, 3H), 3.78 (s, 3H), 3.48 (d, *J* = 2.0 Hz, 2H), 1.89 – 1.74 (m, 2H), 1.04 (t, *J* = 7.4 Hz, 3H). ^{13}C NMR (101 MHz, $CDCl_3$) δ (ppm) 192.72, 161.03, 159.17, 158.90, 154.10, 149.75, 147.57, 139.03, 138.71, 136.44, 133.97, 130.61, 129.76, 129.64, 127.94, 124.87, 116.88, 114.72, 101.60, 101.47, 97.52, 69.54, 61.12, 60.44, 56.30, 55.66, 55.47, 28.66, 22.54, 10.50. MS (ESI): calcd. for $C_{32}H_{34}O_7$, $[M+H]^+$, 531.2383; found 531.2379.

2-((E)-2,4-dimethoxy-6-((E)-4-propoxystyryl)benzylidene)-5,6-dimethoxy-2,3-dihydro-1H-inden-1-one (**PIF_8**). Yellow solid, Yield: 40.2%, m.p.: 192-194°C. ^{1}H NMR (400 MHz, $CDCl_3$) δ (ppm) 7.79 (s, 1H), 7.42 – 7.34 (m, 3H), 7.12 – 6.99 (m, 2H), 6.85 (dd, *J* = 5.6, 3.2 Hz, 3H), 6.80 (s, 1H), 6.45 (d, *J* = 2.2 Hz, 1H), 3.96 (s, 3H), 3.92 (dd, *J* = 7.7, 3.7 Hz, 8H), 3.84 (s, 3H), 3.48 (d, *J* = 1.7 Hz, 2H), 1.88 – 1.75 (m, 2H), 1.04 (t, *J* = 7.4 Hz, 3H). ^{13}C NMR (101 MHz, $CDCl_3$) δ (ppm) 192.59, 160.89, 159.17, 158.95, 155.19, 149.35, 145.39, 139.72, 138.49, 131.45, 130.24, 129.63, 128.62, 127.95, 124.80, 117.19, 114.75, 107.26, 105.05, 101.30, 97.47, 69.54, 56.20, 56.17, 55.64, 55.46, 31.52, 22.55, 10.51. MS (ESI): calcd. for $C_{31}H_{32}O_6$, $[M + H]^+$, 501.2272; found 501.2267

2-((E)-2,4-dimethoxy-6-((E)-4-propoxystyryl)benzylidene)-5,7-dimethoxy-2,3-dihydro-1H-inden-1-one (**PIF_9**). Yellow solid, Yield: 44.8%, m.p.: 167-169°C. ^{1}H NMR (400 MHz, $CDCl_3$) δ (ppm) 7.71 (s, 1H), 7.38 (d, *J* = 8.7 Hz, 2H), 7.04 (s, 2H), 6.85 (d, *J* = 8.6 Hz, 3H), 6.48 – 6.36 (m, 2H), 6.33 (d, *J* = 1.4 Hz, 1H), 3.96 (d, *J* = 8.1 Hz, 3H), 3.95 – 3.89 (m, 5H), 3.84 (d, *J* = 7.3 Hz, 6H), 3.47 (s, 2H), 1.87 – 1.76 (m, 2H), 1.04 (t, *J* = 7.4 Hz, 3H). ^{13}C NMR (101 MHz, $CDCl_3$) δ (ppm) 190.29, 166.65, 160.73, 160.09, 159.12, 158.79, 154.98, 139.76, 138.52, 130.12, 129.67, 127.94, 127.50, 124.72, 121.02, 117.28, 114.73, 101.45, 101.20, 97.53, 97.35, 69.54, 55.83, 55.69, 55.63, 55.44, 32.11, 22.56, 10.52. MS (ESI): calcd. for $C_{31}H_{32}O_6$, $[M + H]^+$, 501.2272; found 501.2274

2-((E)-2,4-dimethoxy-6-((E)-4-methoxystyryl)benzylidene)-4,5,6-trimethoxy-2,3-dihydro-1H-inden-1-one (**PIF_10**). Yellow solid, Yield: 62.1%, m.p.: 178-181°C. ^{1}H NMR (400 MHz, $CDCl_3$) δ (ppm)7.82 (s, 1H), 7.40 (d, *J* = 8.8 Hz, 2H), 7.21 (s, 1H), 7.05 (q, *J* = 16.2 Hz, 2H), 6.90 – 6.82 (m, 3H), 6.46 (d, *J* = 2.2 Hz, 1H), 3.99 – 3.89 (m,

9H), 3.85 (s, 3H), 3.81 (s, 3H), 3.78 (s, 3H), 3.48 (d, J = 2.0 Hz, 2H). ^{13}C NMR (101 MHz, CDCl3) δ (ppm)192.70, 161.04, 159.57, 158.90, 154.12, 149.75, 147.57, 139.04, 138.67, 136.43, 133.97, 130.53, 129.86, 129.73, 127.96, 125.04, 116.90, 114.17, 101.61, 101.51, 97.56, 61.12, 60.44, 56.30, 55.66, 55.47, 55.31, 28.66. MS (ESI): calcd. for $C_{30}H_{30}O_7$, [M + H]$^+$, 503.2064; found 503.2060

2-((E)-2,4-dimethoxy-6-((E)-4-methoxystyryl)benzylidene)-5,7-dimethoxy-2,3-dihydro-1H-inden-1-one (**PIF_11**). Yellow solid, Yield: 39.6%, Mp: 197-199°C. ^{1}H NMR (400 MHz, $CDCl_3$) δ (ppm)7.72 (s, 1H), 7.40 (d, J = 8.6 Hz, 2H), 7.05 (s, 2H), 6.85 (dd, J = 8.3, 5.4 Hz, 3H), 6.48 – 6.38 (m, 2H), 6.33 (s, 1H), 3.97 (s, 3H), 3.93 (d, J = 4.4 Hz, 3H), 3.87 – 3.80 (m, 9H), 3.47 (d, J = 0.9 Hz, 2H). ^{13}C NMR (101 MHz, $CDCl_3$) δ (ppm) 190.29, 166.66, 160.73, 160.10, 159.52, 158.79, 154.98, 139.77, 138.47, 130.04, 129.89, 127.97, 127.50, 124.90, 121.02, 117.30, 114.18, 101.44, 101.21, 97.56, 97.35, 55.84, 55.69, 55.64, 55.45, 55.33, 32.11. MS (ESI): calcd. for $C_{29}H_{28}O_6$, [M + H]$^+$, 473.1959; found 473.1955

2-((E)-2,4-dimethoxy-6-((E)-4-(prop-2-yn-1-yloxy)styryl)benzylidene)-4,5,6-trimethoxy-2,3-dihydro-1H-inden-1-one (**PIF_12**). Yellow solid, Yield: 45.3%, m.p.: 180-181°C. ^{1}H NMR (400 MHz, $CDCl_3$) δ (ppm)7.81 (s, 1H), 7.41 (d, J = 8.8 Hz, 2H), 7.21 (s, 1H), 7.11 – 6.99 (m, 2H), 6.93 (d, J = 8.7 Hz, 2H), 6.84 (d, J = 2.2 Hz, 1H), 6.47 (d, J = 2.2 Hz, 1H), 4.70 (d, J = 2.4 Hz, 2H), 3.99 – 3.90 (m, 9H), 3.85 (s, 3H), 3.78 (s, 3H), 3.48 (d, J = 1.9 Hz, 2H), 2.53 (t, J = 2.4 Hz, 1H). ^{13}C NMR (101 MHz, $CDCl_3$) δ(ppm) 192.70, 161.04, 158.89, 157.42, 154.13, 149.75, 147.59, 139.07, 138.55, 136.44, 133.95, 130.74, 130.34, 129.69, 127.93, 125.54, 116.95, 115.15, 114.97, 101.62, 97.63, 78.35, 75.69, 61.12, 60.46, 56.31,55.81, 55.67, 55.48, 28.64. MS (ESI): calcd. for $C_{32}H_{30}O_7$, [M + H]$^+$, 527.2064 found 527.2061

2-((E)-2,4-dimethoxy-6-((E)-4-(prop-2-yn-1-yloxy)styryl)benzylidene)-5,7-dimethoxy-2,3-dihydro-1H-inden-1-one (**PIF_13**). Yellow solid, Yield: 51.9%, m.p.: 155-158°C.

^{1}H NMR (400 MHz, $CDCl_3$) δ (ppm)7.70 (s, 1H), 7.41 (d, *J* = 8.8 Hz, 2H), 7.06 (s, 2H), 6.93 (d, *J* = 8.8 Hz, 2H), 6.83 (d, *J* = 2.1 Hz, 1H), 6.48 – 6.37 (m, 2H), 6.33 (d, *J* = 1.4 Hz, 1H), 4.72 (dd, *J* = 13.6, 2.3 Hz, 2H), 3.97 (s, 3H), 3.92 (s, 3H), 3.84 (d, *J* = 7.4 Hz, 6H), 3.46 (s, 2H), 2.54 (t, *J* = 2.3 Hz, 1H).^{13}C NMR (101 MHz, $CDCl_3$) δ (ppm) 190.25, 166.67, 160.74, 160.10, 158.78, 157.40, 154.95, 139.82, 138.37, 130.78, 129.87, 127.93, 127.42, 125.40, 121.01, 117.36, 115.15, 101.47, 101.31, 97.65, 97.36, 78.38, 75.68, 60.41, 55.83, 55.69, 55.64, 55.45, 32.09, 21.07, 14.21. MS (ESI): calcd. for $C_{31}H_{28}O_6$, $[M + H]^+$, 497.1959; found 497.1960

2-((E)-2,4-dimethoxy-6-((E)-4-((4-methylbenzyl)oxy)styryl)benzylidene)-4,5,6-trimethoxy-2,3-dihydro-1H-inden-1-one (**PIF_14**). Yellow solid, Yield: 41.6%, m.p.: 211-213°C. ^{1}H NMR (400 MHz, $CDCl_3$) δ 7.86 – 7.77 (m, 1H), 7.39 (d, *J* = 8.8 Hz, 2H), 7.32 (d, *J* = 8.0 Hz, 2H), 7.21 (s, 2H), 7.19 (s, 1H), 7.04 (q, *J* = 16.2 Hz, 2H), 6.92 (t, *J* = 5.8 Hz, 2H), 6.84 (d, *J* = 2.2 Hz, 1H), 6.46 (d, *J* = 2.2 Hz, 1H), 5.03 (s, 2H), 4.00 – 3.88 (m, 9H), 3.85 (s, 3H), 3.77 (s, 3H), 3.49 (t, *J* = 7.3 Hz, 2H), 2.37 (s, 3H). ^{13}C NMR (101 MHz, $CDCl_3$) δ (ppm)192.71, 190.68, 161.03, 158.90, 158.83, 154.11, 149.75, 147.58, 139.05, 138.66, 137.82, 136.44, 133.97, 133.72, 130.53, 130.01, 129.73, 129.29, 128.38, 127.96, 127.61, 125.62, 125.09, 116.91, 115.11, 101.61, 101.50, 97.56, 69.96, 61.12, 60.45, 56.31, 55.66, 55.47, 28.66, 21.21. MS (ESI): calcd. for $C_{37}H_{36}O_7$, $[M + H]^+$, 593.2534; found 593.2537

2-((E)-2-((E)-4-(benzyloxy)styryl)-4,6-dimethoxybenzylidene)-4,5,6-trimethoxy-2,3-dihydro-1H-inden-1-one (**PIF_15**). Yellow solid, Yield: 37.8%, m.p.: 140-143°C. ^{1}H NMR (400 MHz, $CDCl_3$) δ (ppm) 7.82 (s, 1H), 7.43 (t, *J* = 5.9 Hz, 3H), 7.41 – 7.37 (m, 3H), 7.36 – 7.31 (m, 1H), 7.21 (s, 1H), 7.10 – 7.02 (m, 2H), 6.92 (t, *J* = 7.8 Hz, 2H), 6.85 (d, *J* = 2.2 Hz, 1H), 6.46 (d, *J* = 2.2 Hz, 1H), 5.07 (s, 2H), 4.04 – 3.91 (m, 9H), 3.86 (d, *J* = 5.6 Hz, 3H), 3.78 (d, *J* = 8.8 Hz, 3H), 3.48 (d, *J* = 1.9 Hz, 2H). ^{13}C NMR (101 MHz, CDCl3) δ (ppm) 192.74, 161.03, 158.89, 158.74, 154.10, 149.74, 147.57, 139.05, 138.63, 136.76, 136.45, 133.96, 130.48, 130.09, 129.74, 128.62, 128.04,

127.99, 127.49, 125.15, 116.90, 115.10, 101.60, 101.47, 97.56, 70.01, 61.14, 60.47, 56.32, 55.67, 55.49, 28.66. MS (ESI): calcd. for $C_{36}H_{34}O_7$, $[M + H]^+$, 579.2477, found 579.2425

2-((E)-2-((E)-4-isobutoxystyryl)-4,6-dimethoxybenzylidene)-5,6-dimethoxy-2,3-dihydro-1H-inden-1-one (**PIF_16**). Yellow solid, Yield: 40.1%, m.p.: 145-148°C. ^{1}H NMR (400 MHz, $CDCl_3$) δ (ppm)7.79 (t, *J* = 1.9 Hz, 1H), 7.37 (dd, *J* = 8.7, 5.9 Hz, 3H), 7.28 (s, 1H), 6.87 – 6.79 (m, 4H), 6.45 (d, *J* = 2.2 Hz, 1H), 3.94 (dd, *J* = 12.9, 5.1 Hz, 9H), 3.85 (s, 3H), 3.72 (d, *J* = 6.6 Hz, 2H), 3.48 (d, *J* = 1.5 Hz, 2H), 2.07 (dd, *J* = 13.3, 6.7 Hz, 1H), 1.02 (t, *J* = 5.0 Hz, 6H). ^{13}C NMR (101 MHz, $CDCl_3$) δ (ppm) 192.60, 160.89, 159.30, 158.95, 155.41, 155.21, 150.45, 149.39, 145.40, 139.71, 138.49, 131.43, 130.25, 129.91, 129.60, 128.64, 127.93, 124.77, 117.17, 114.77, 107.49, 107.27, 105.06, 104.18, 101.32, 97.47, 74.46, 56.23, 56.10, 55.63, 55.46, 36.54, 31.52, 28.25, 25.59, 19.24. MS (ESI): calcd. for $C_{32}H_{34}O_6$, $[M + H]^+$, 515.2428 found 515.2425

3. ^{1}H NMR, ^{13}C NMR and HR-MS spectra of representative compounds.

^{1}H NMR of compound **PIF_1**

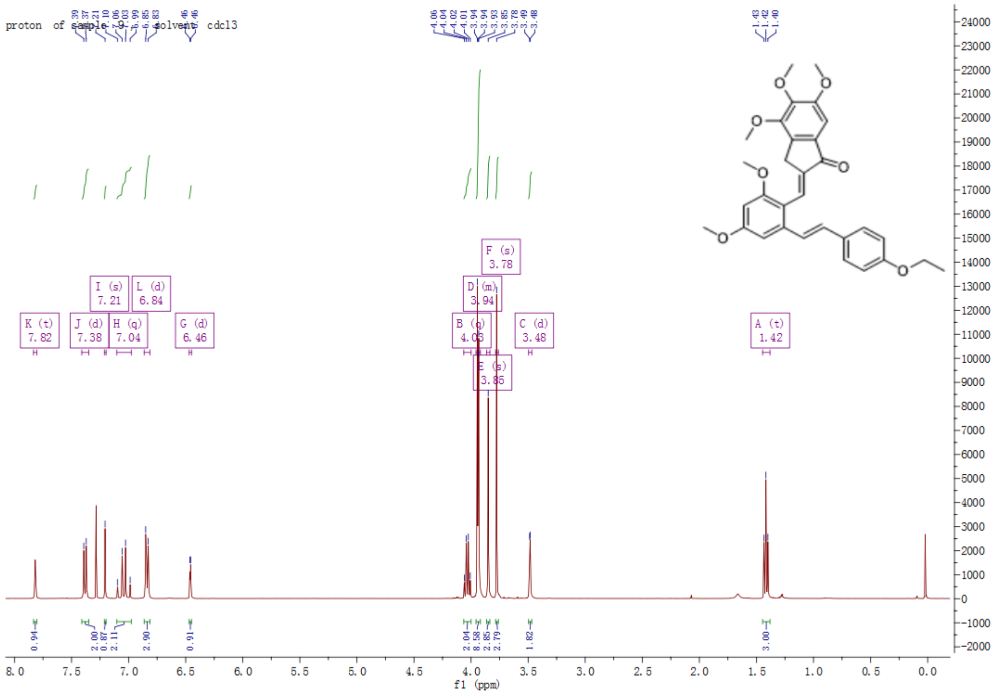

^{13}C NMR of compound **PIF_1**

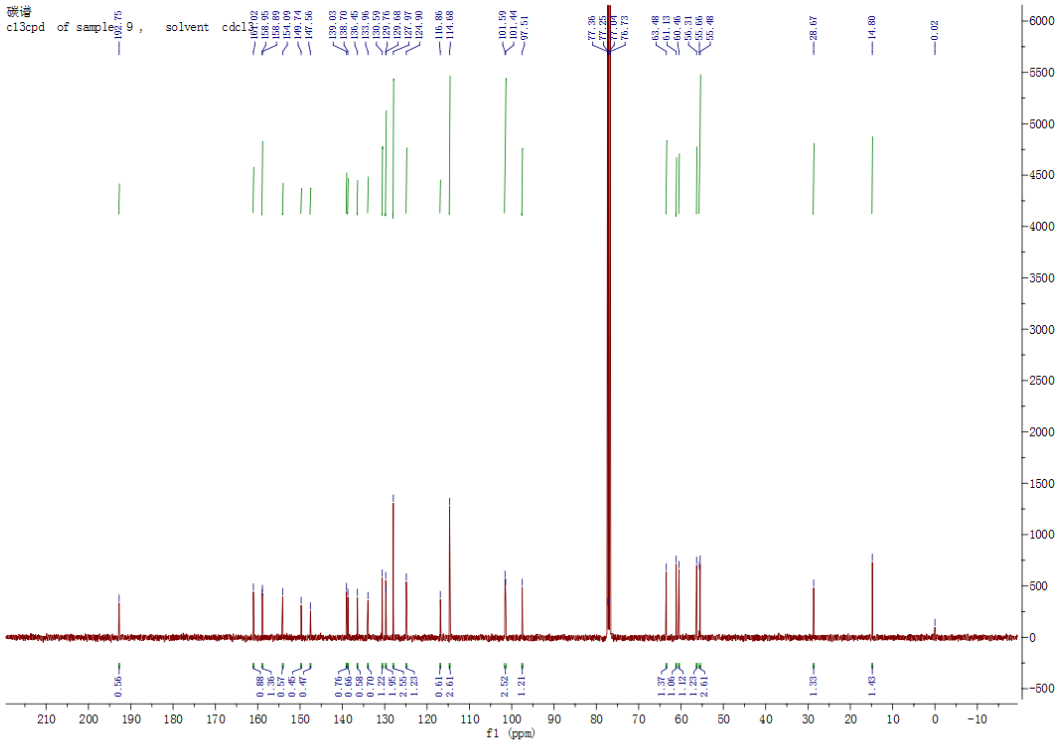

HR-MS of compound **PIF_1**

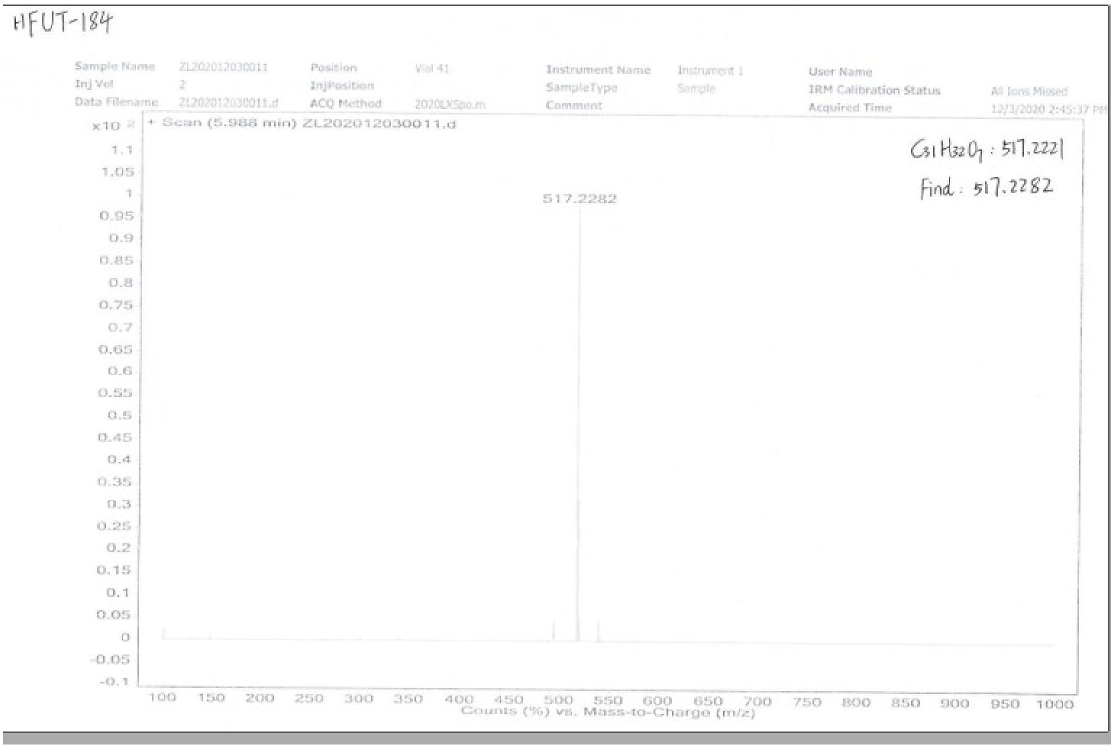

^{1}H NMR of compound **PIF_2**

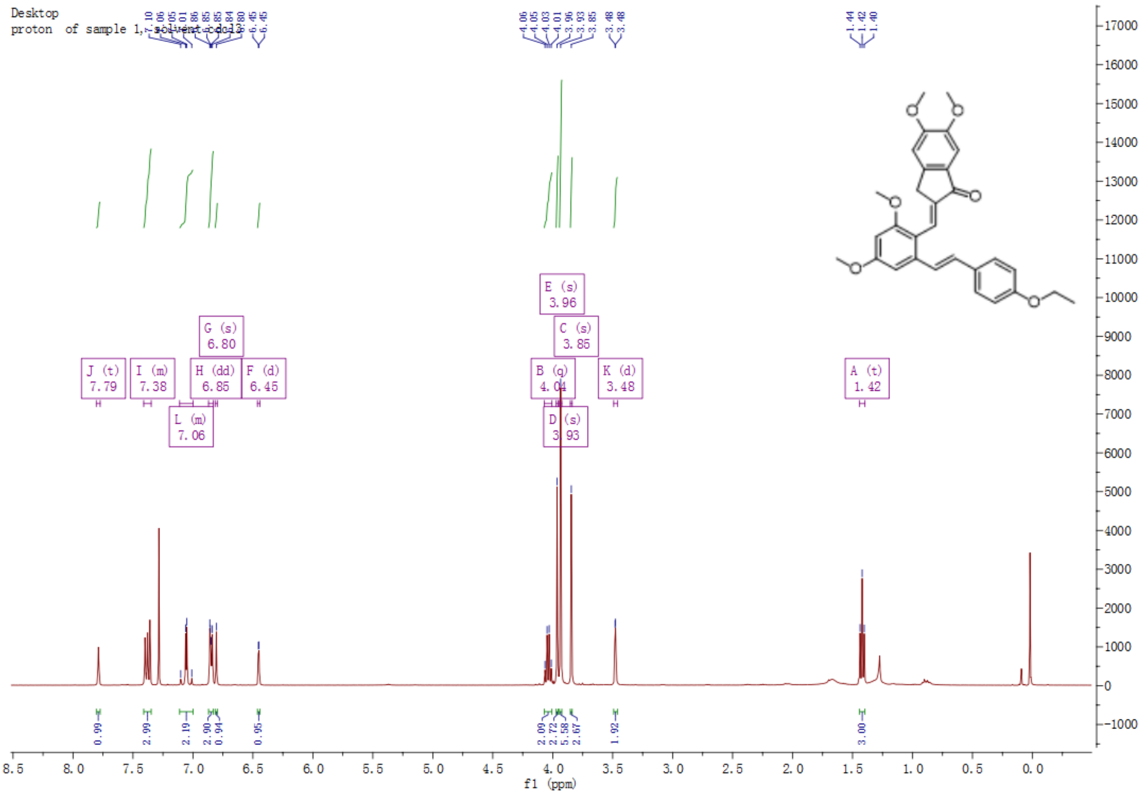

^{13}C NMR of compound **PIF_2**

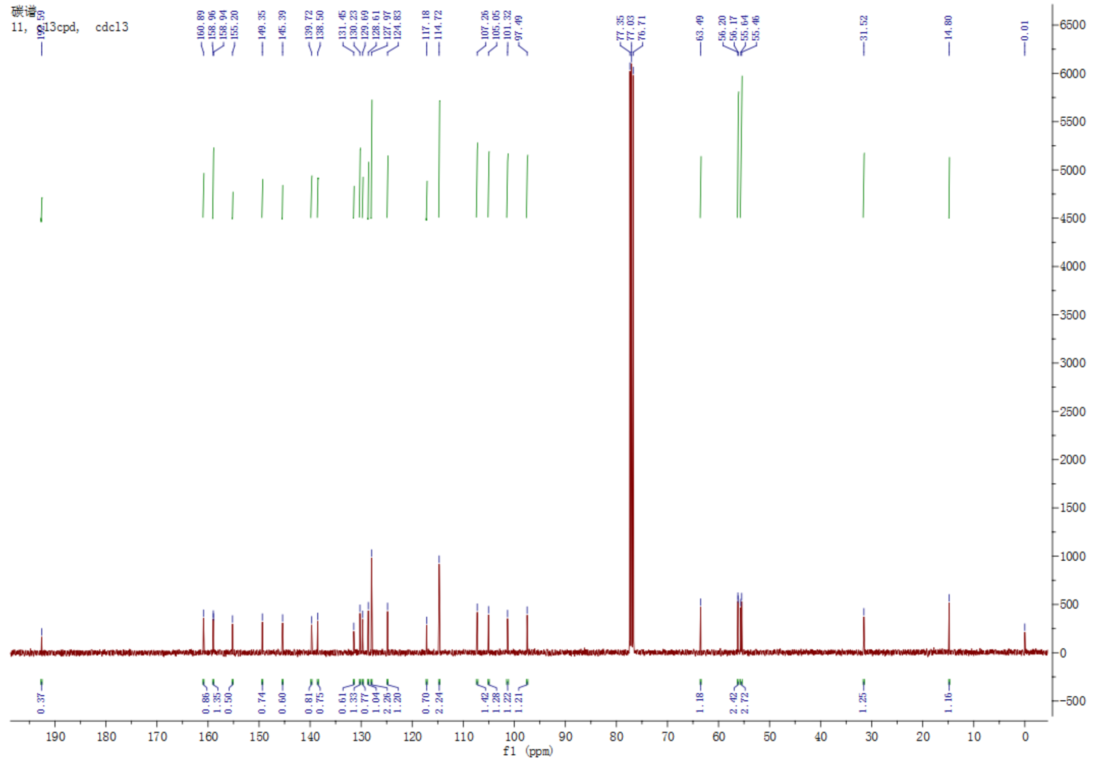

HR-MS of compound **PIF_2**

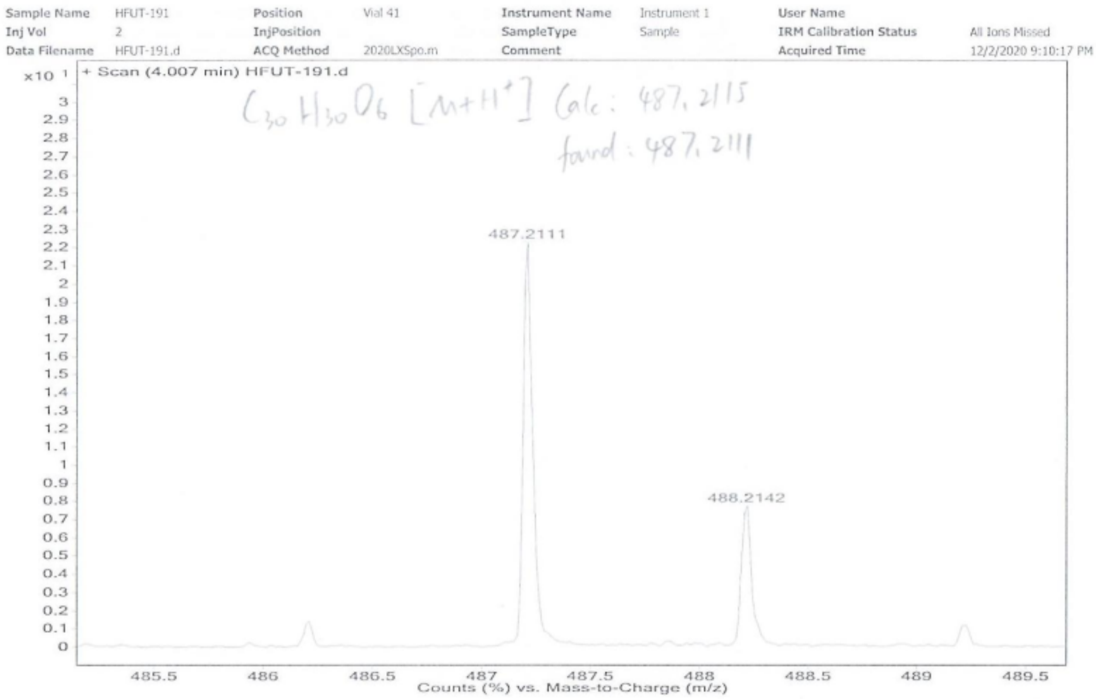

^{1}H NMR of compound **PIF_3**

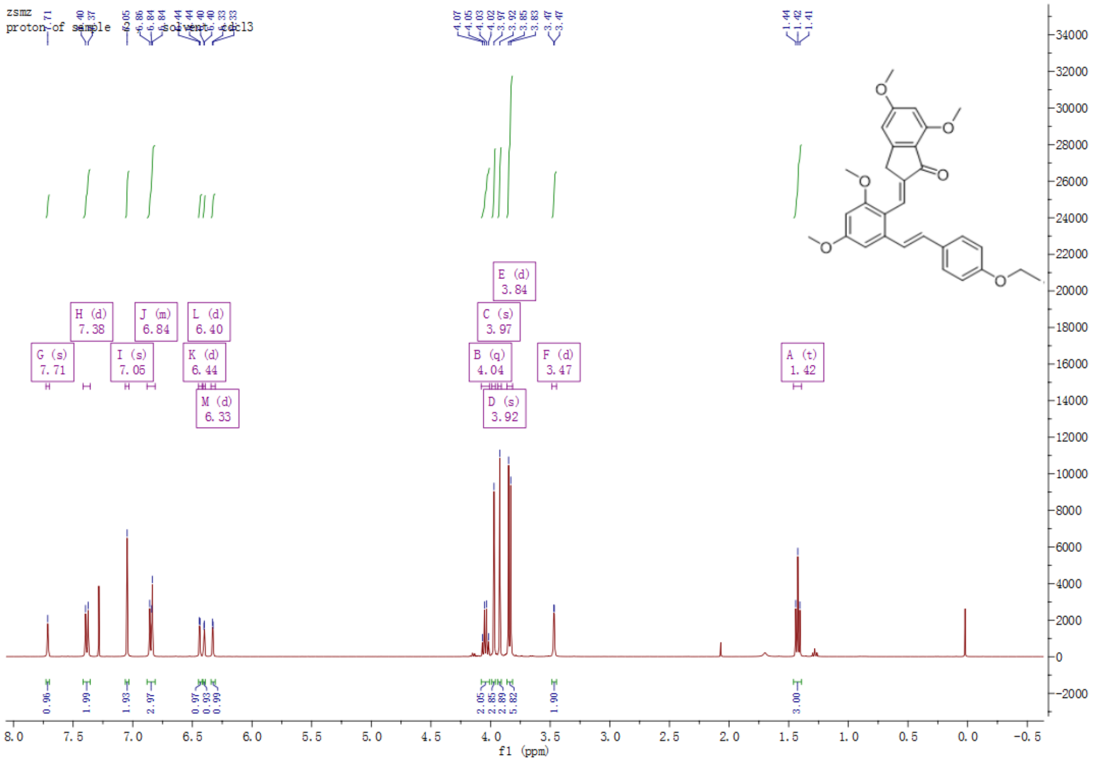

^{13}C NMR of compound **PIF_3**

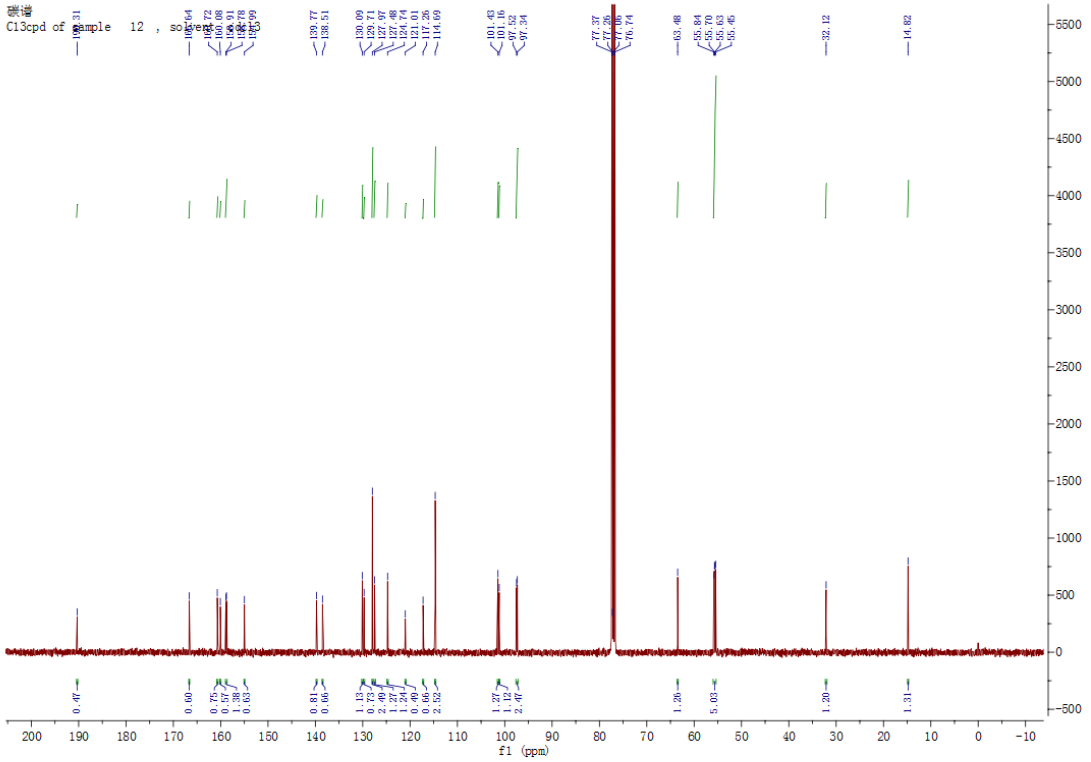

HR-MS of compound **PIF_3**

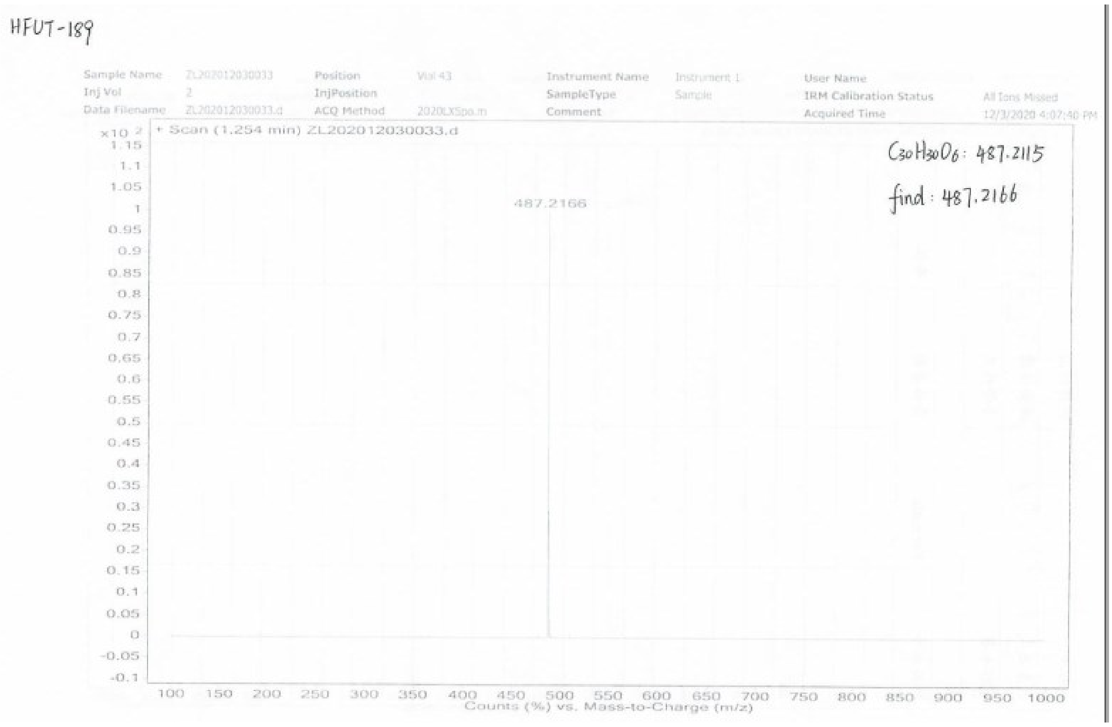

^{1}H NMR of compound **PIF_4**

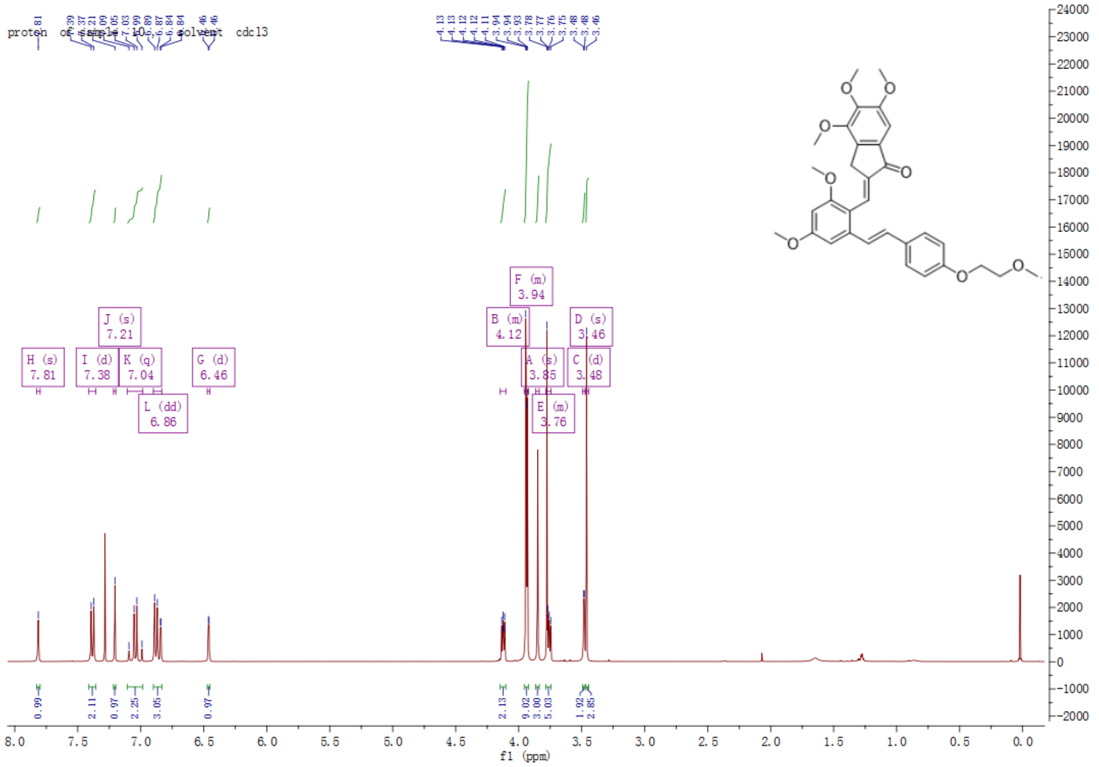

^{13}C NMR of compound **PIF_4**

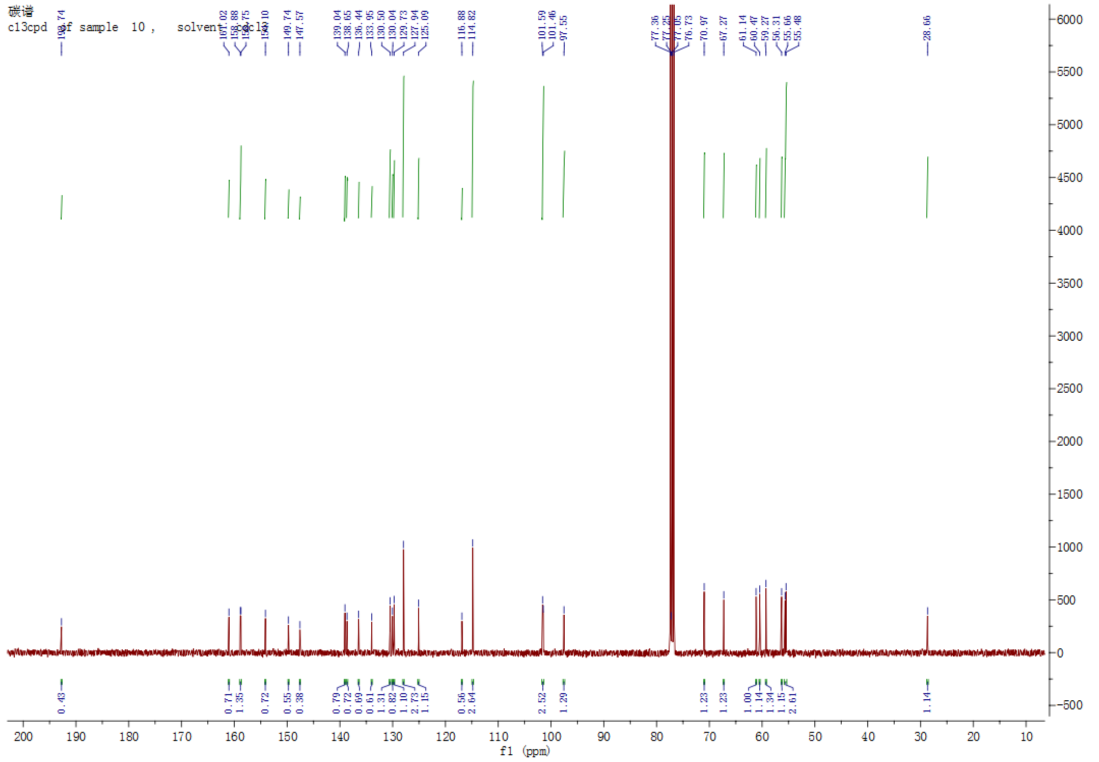

HR-MS of compound **PIF_4**

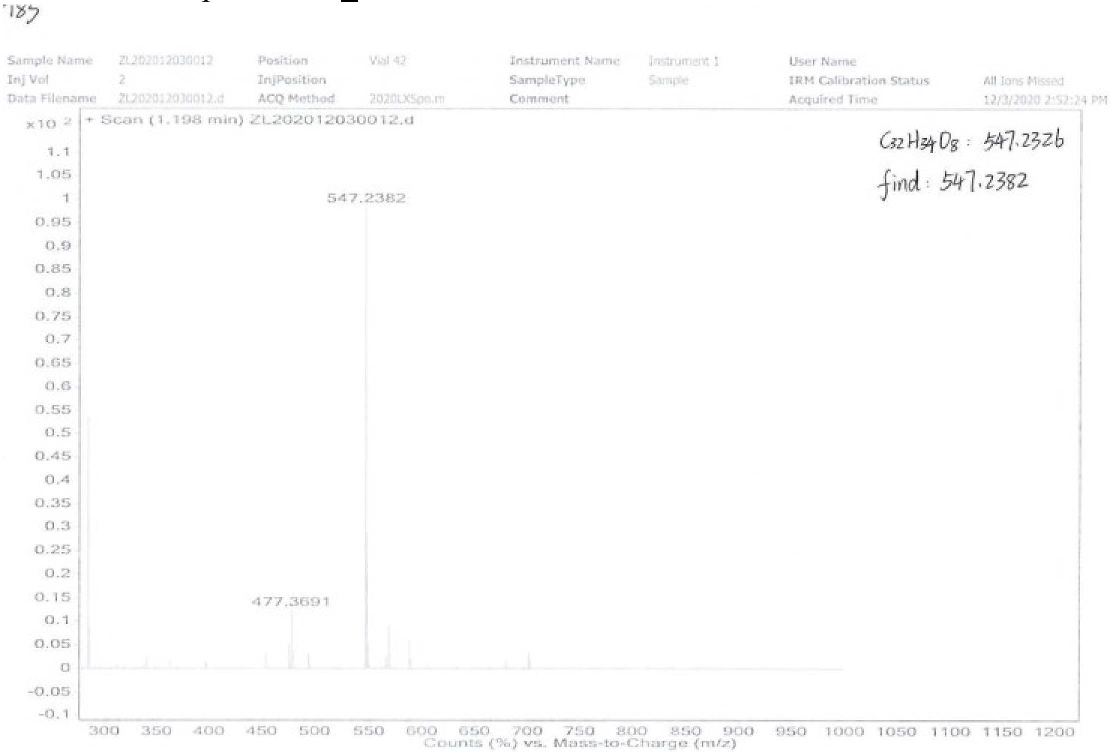

^{1}H NMR of compound **PIF_5**

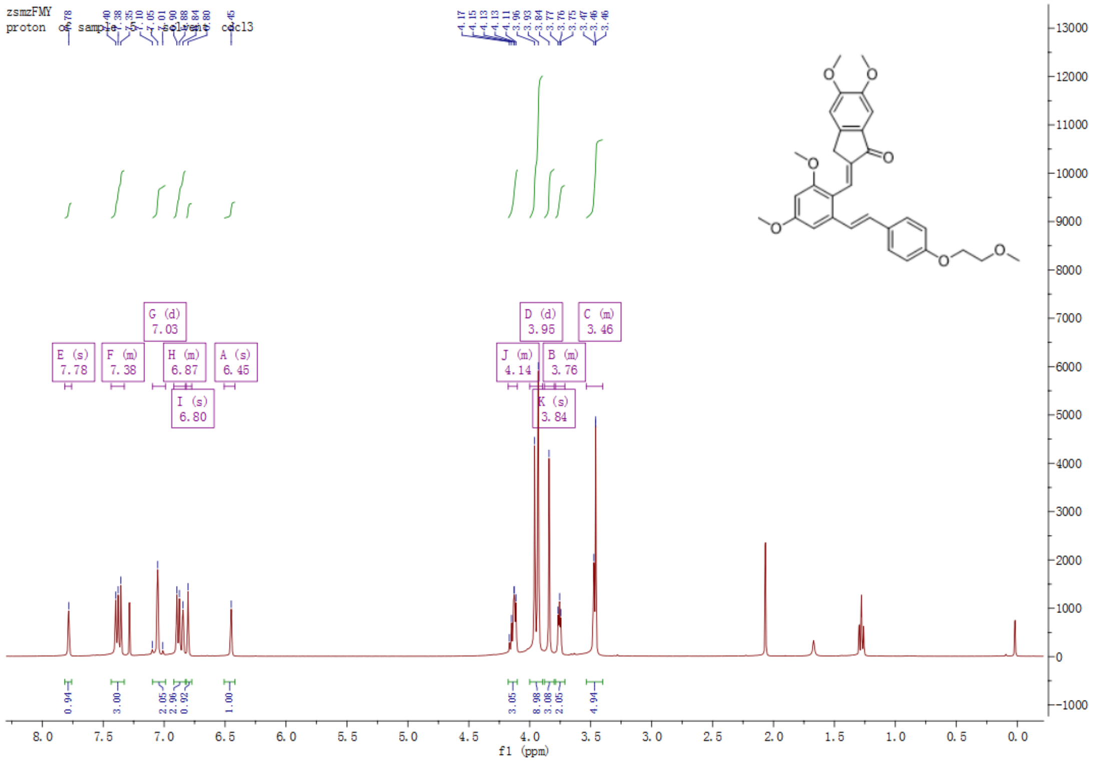

^{13}C NMR of compound **PIF_5**

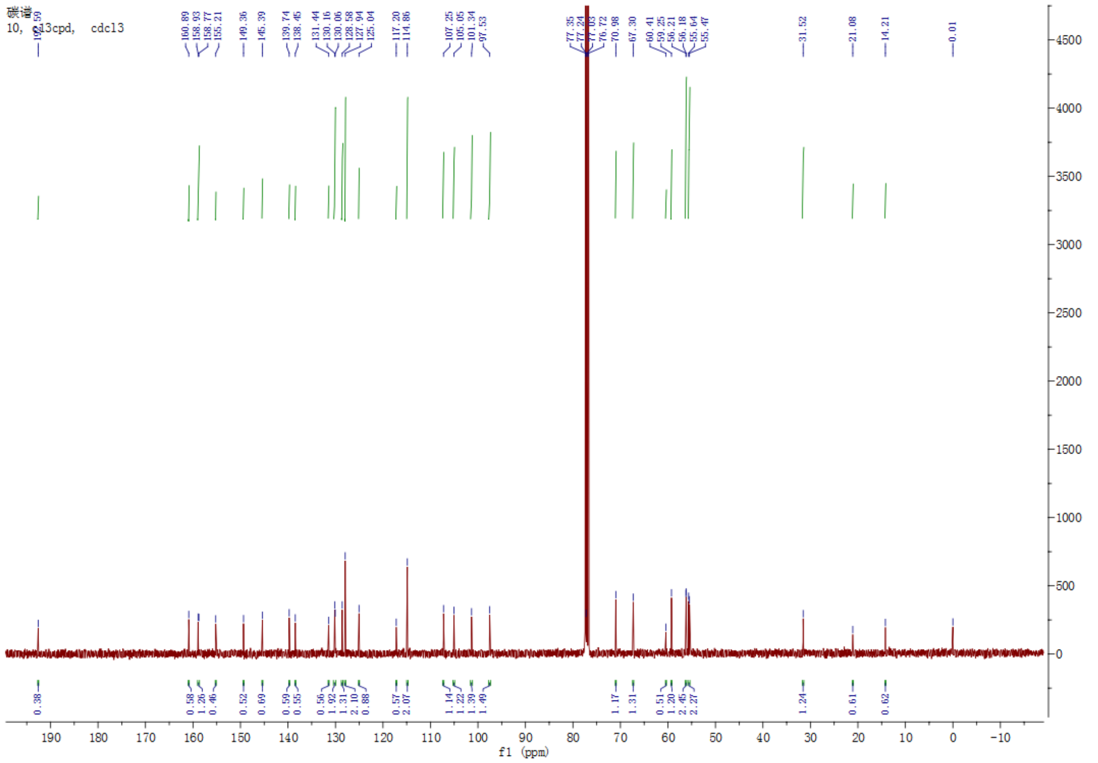

HR-MS of compound **PIF_5**

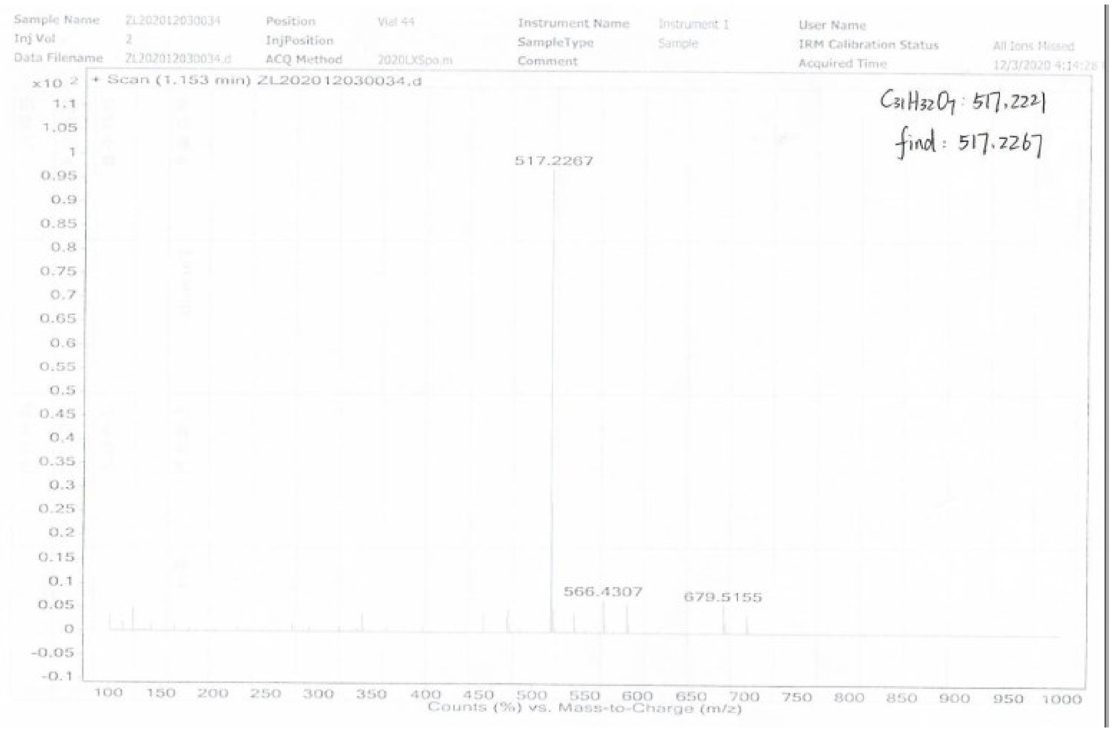

^{1}H NMR of compound **PIF_6**

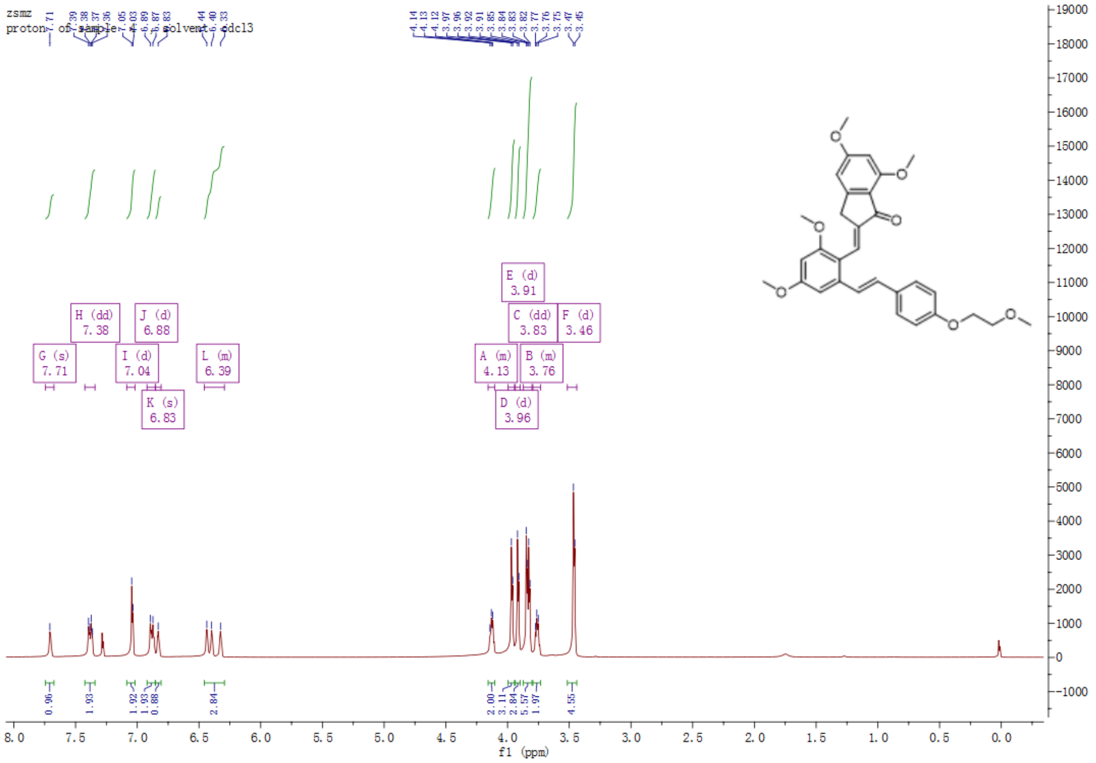

^{13}C NMR of compound **PIF_6**

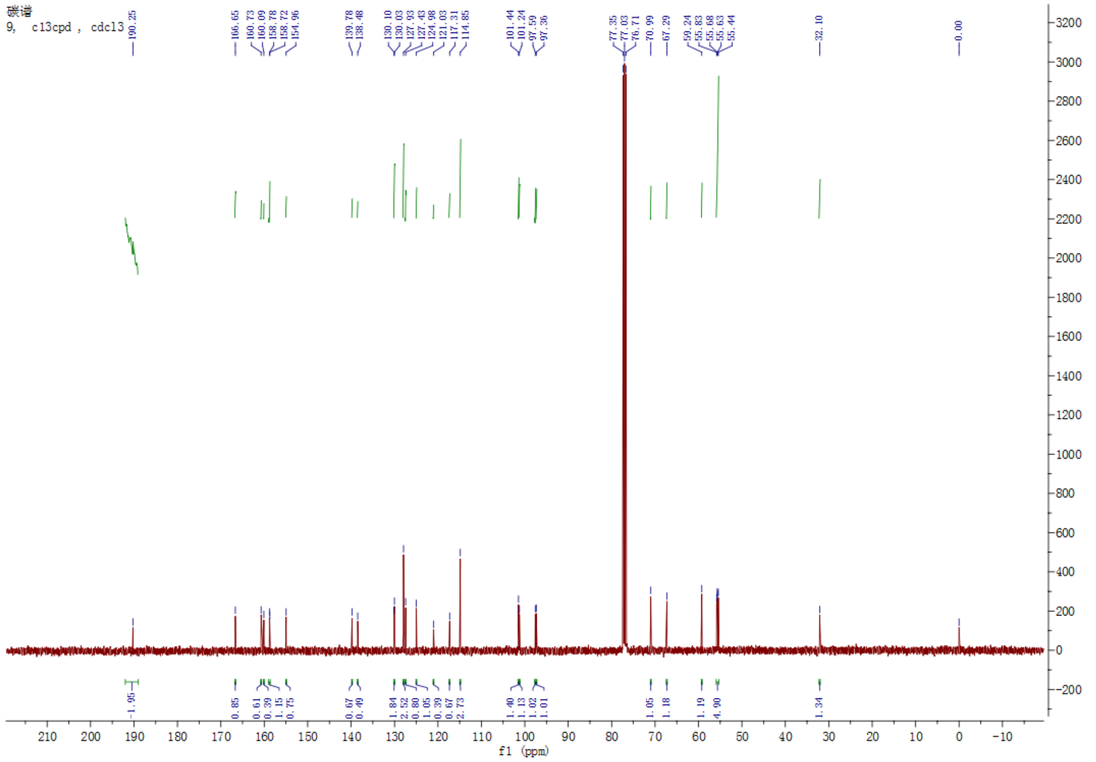

HR-MS of compound **PIF_6**

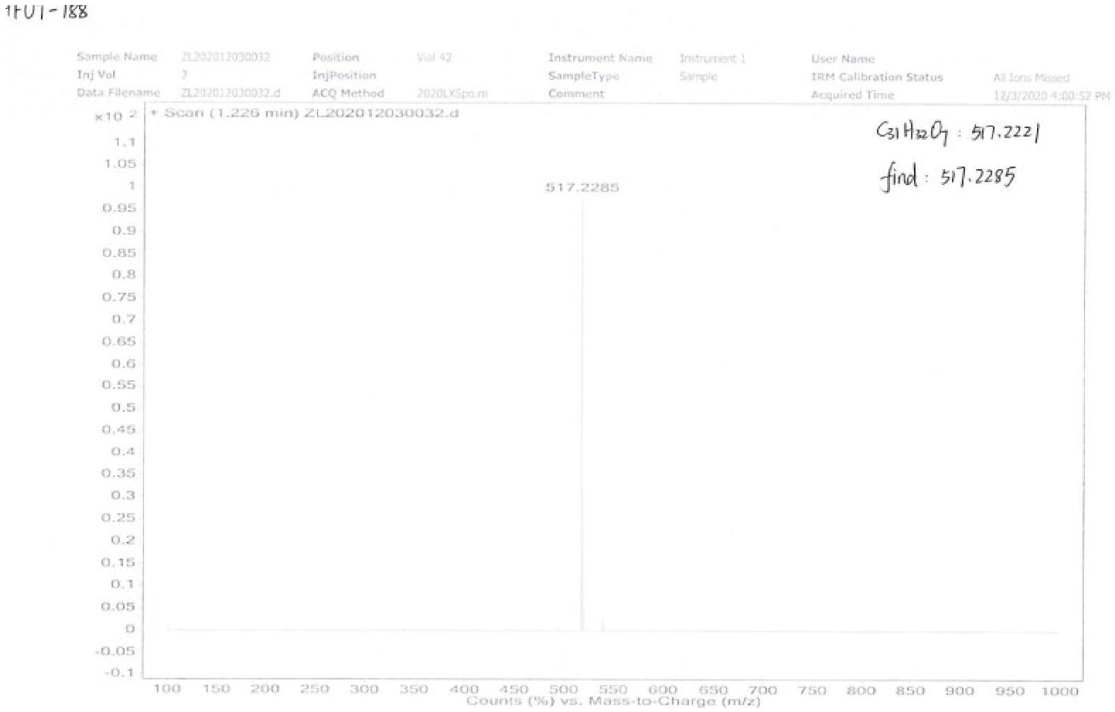

^{1}H NMR of compound **PIF_7**

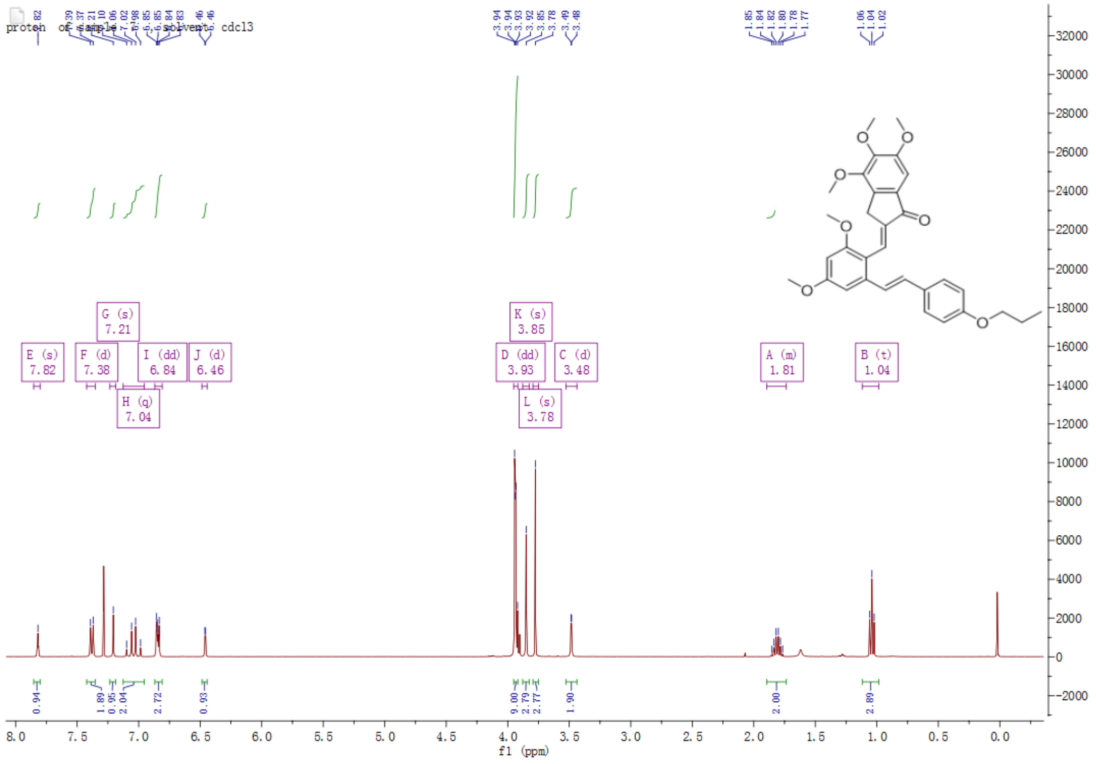

^{13}C NMR of compound **PIF_7**

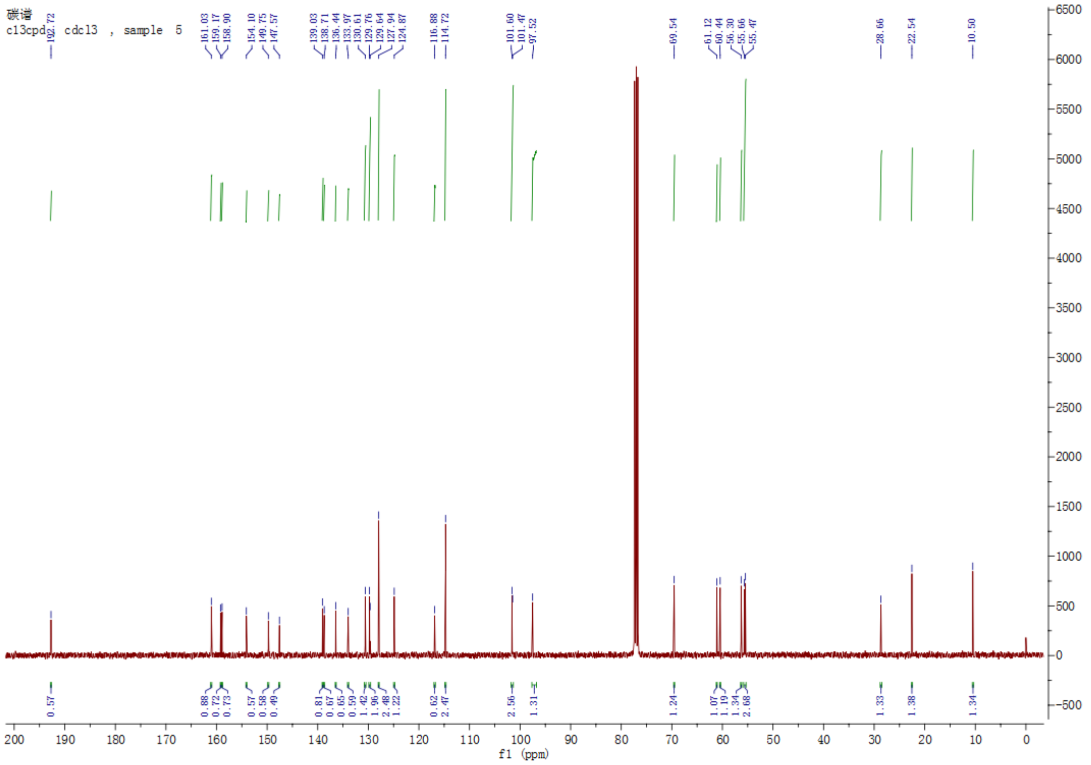

HR-MS of compound **PIF_7**

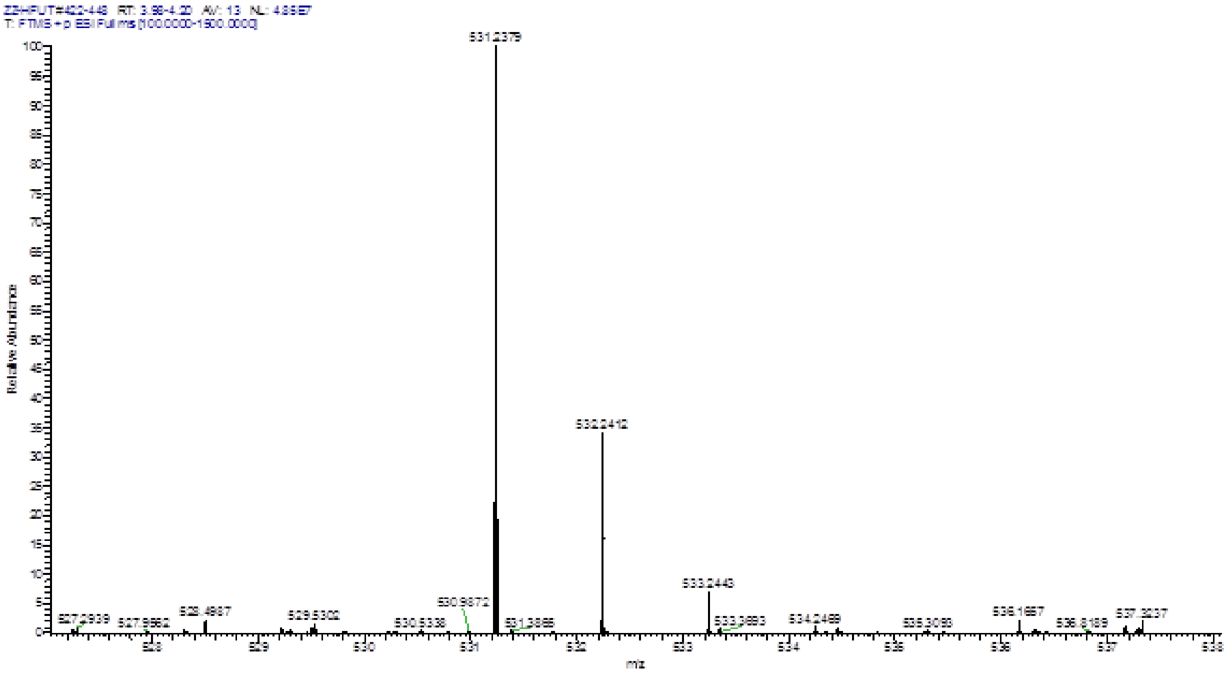

^{1}H NMR of compound **PIF_8**

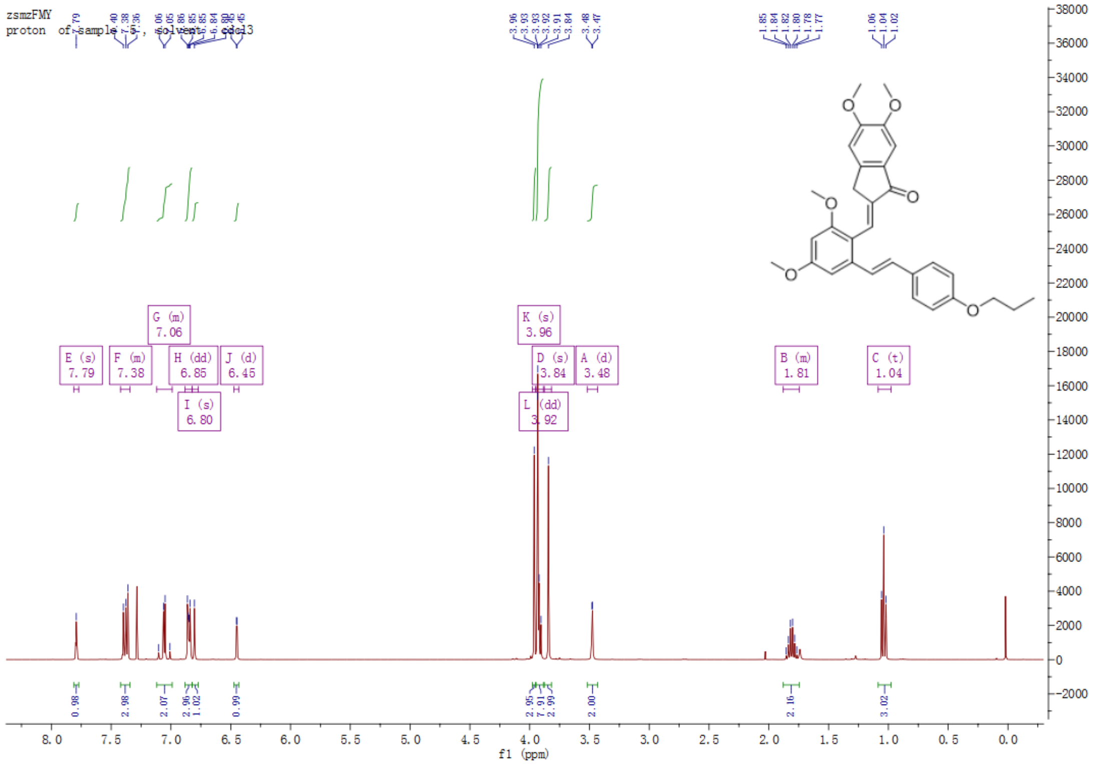

^{13}C NMR of compound **PIF_8**

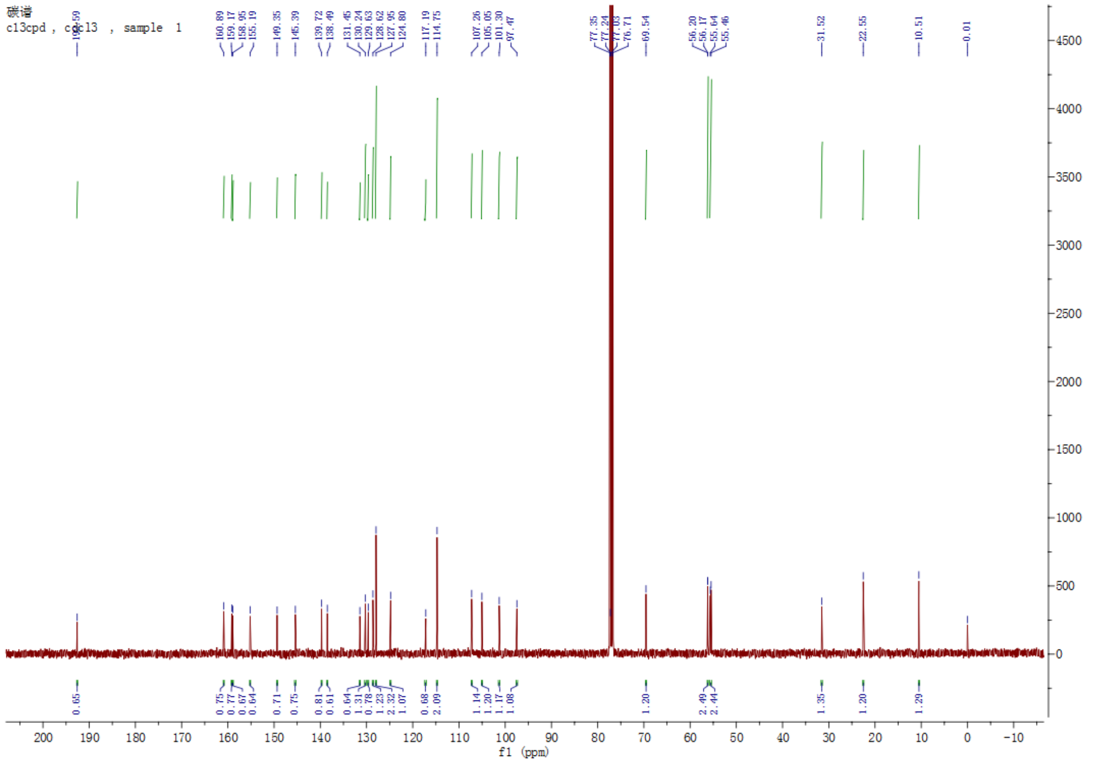

HR-MS of compound **PIF_8**

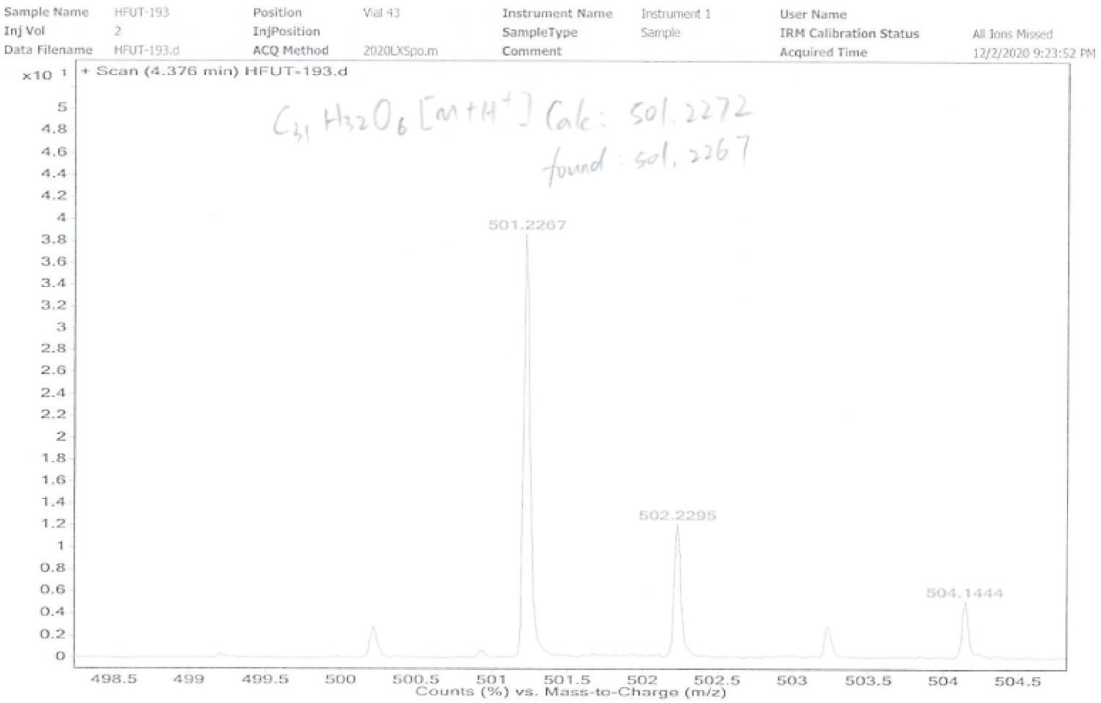

^{1}H NMR of compound **PIF_9**

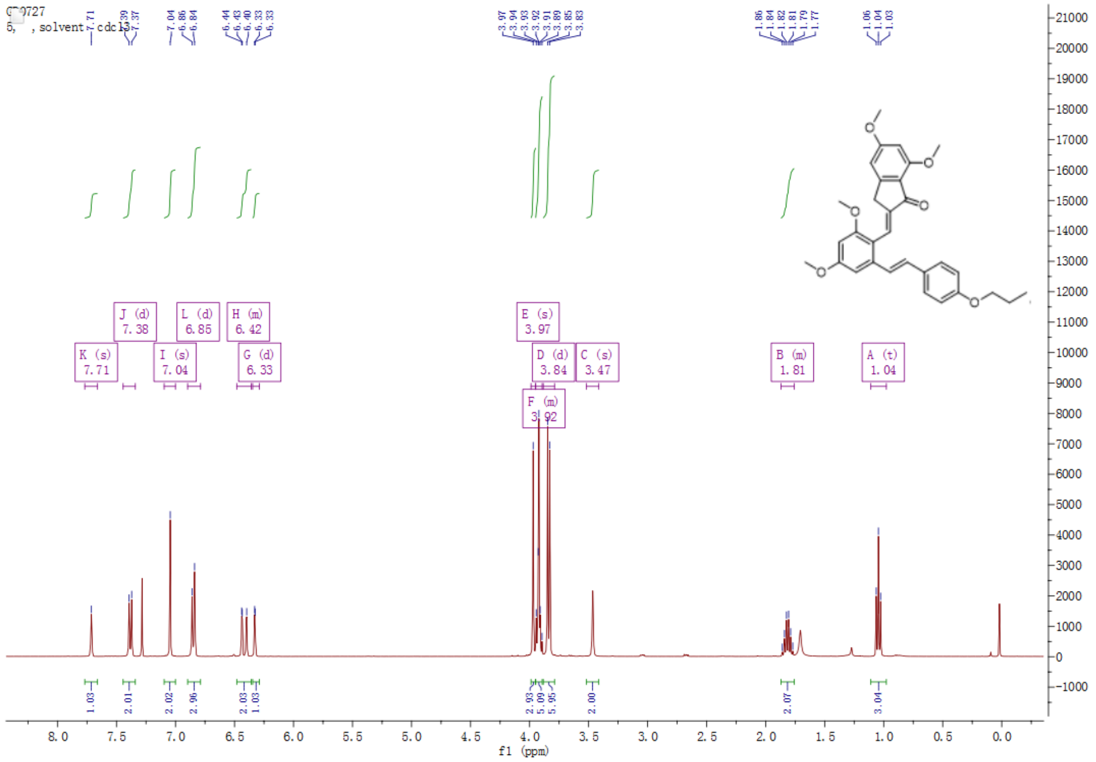

^{13}C NMR of compound **PIF_9**

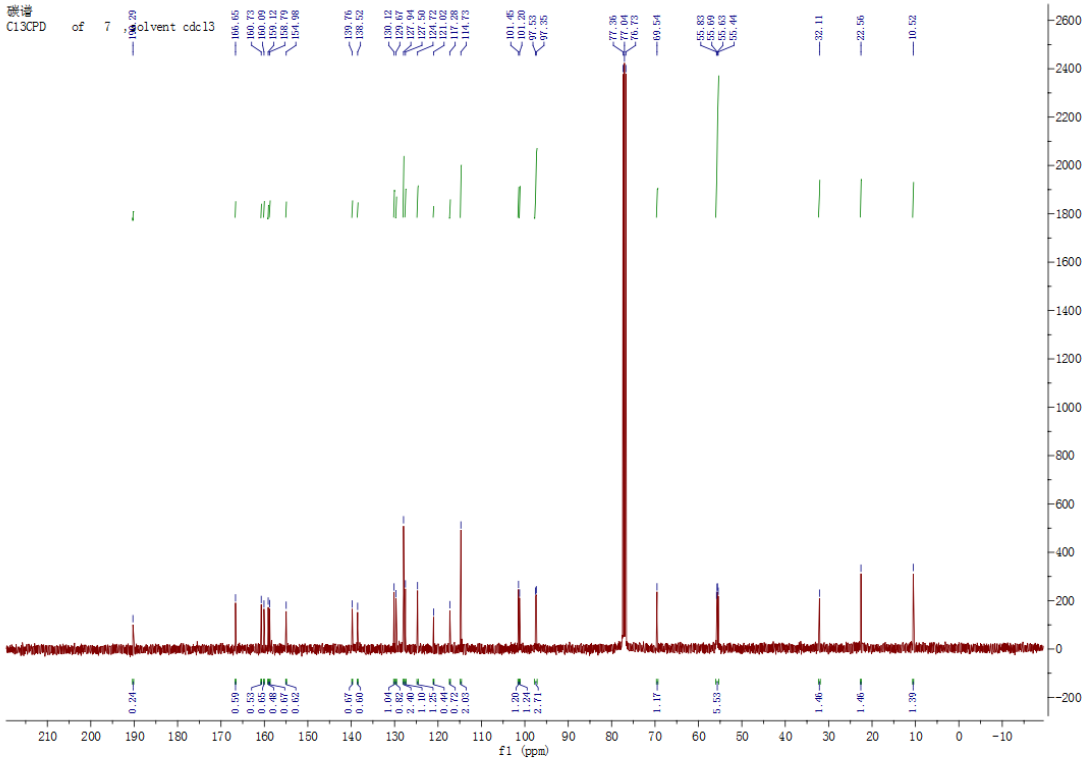

HR-MS of compound **PIF_9**

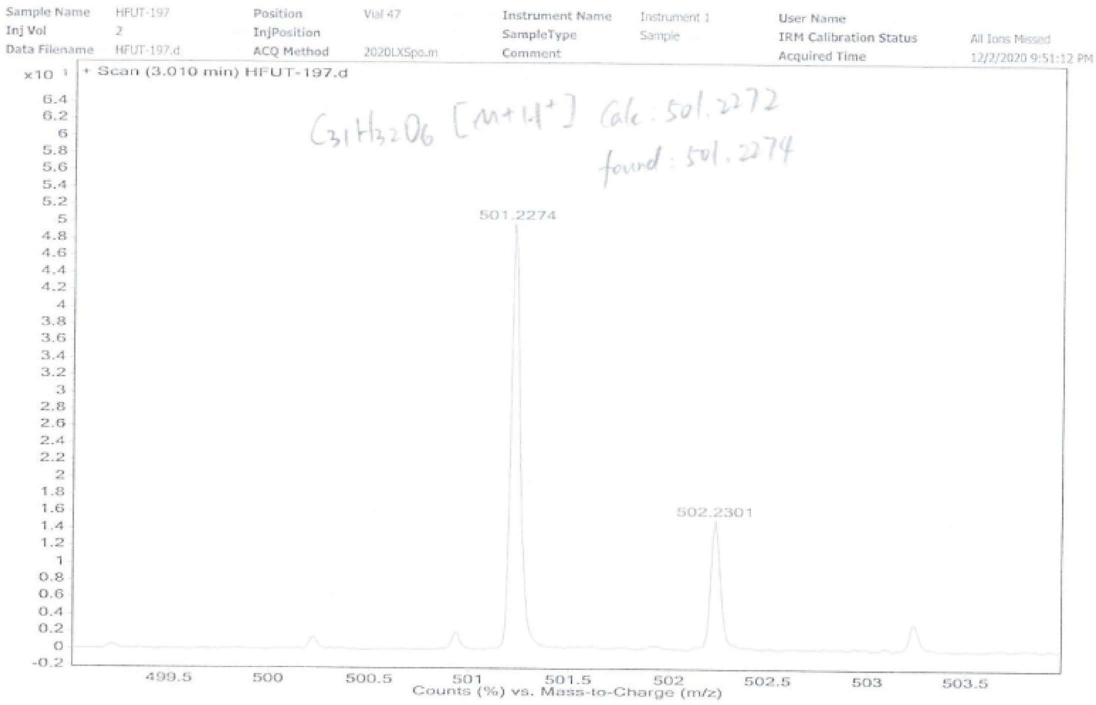

^{1}H NMR of compound **PIF_10**

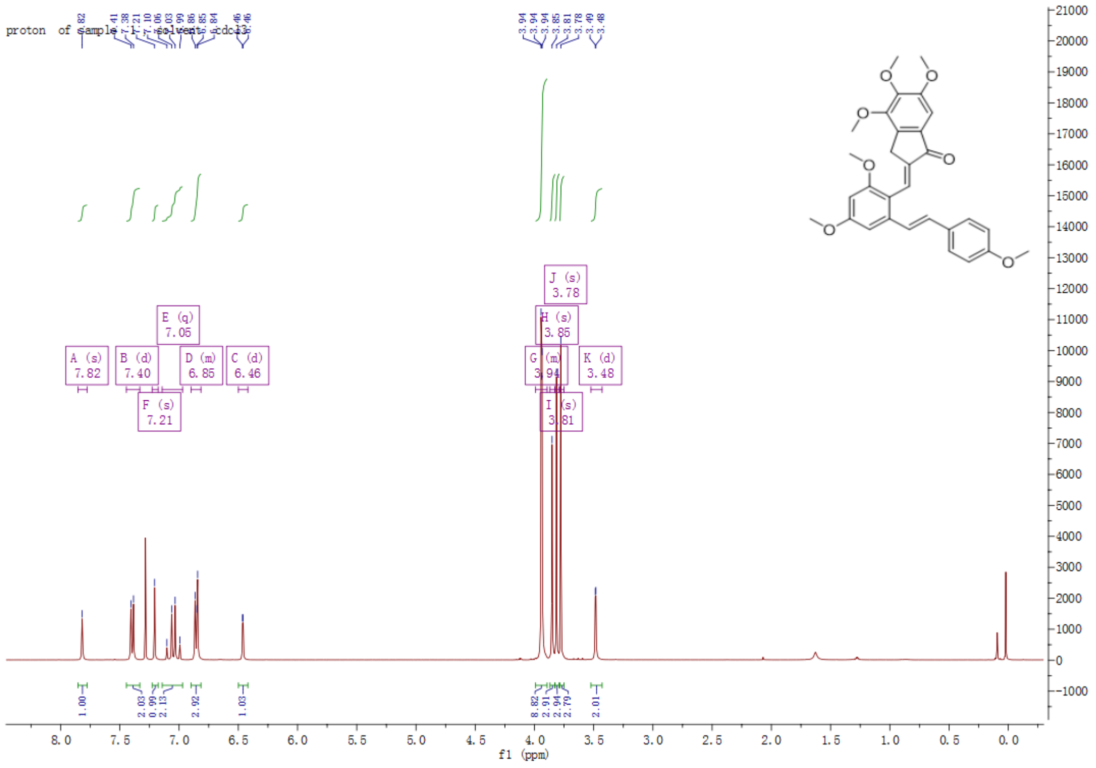

^{13}C NMR of compound **PIF_10**

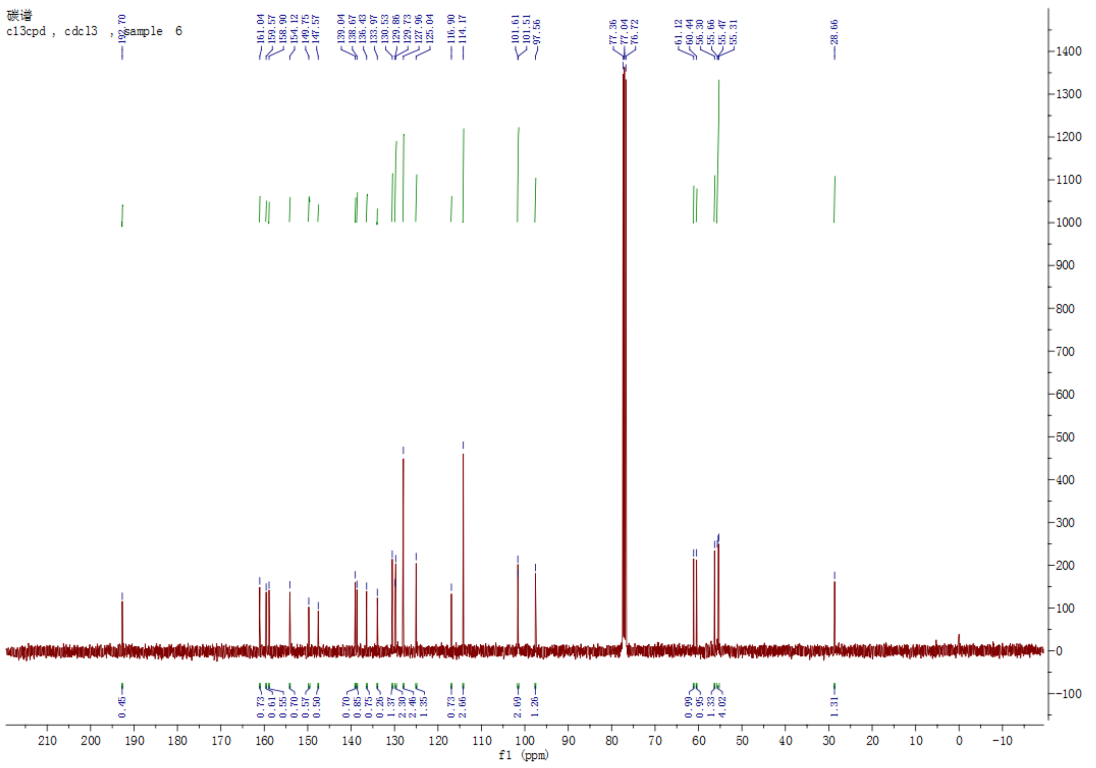

HR-MS of compound **PIF_10**

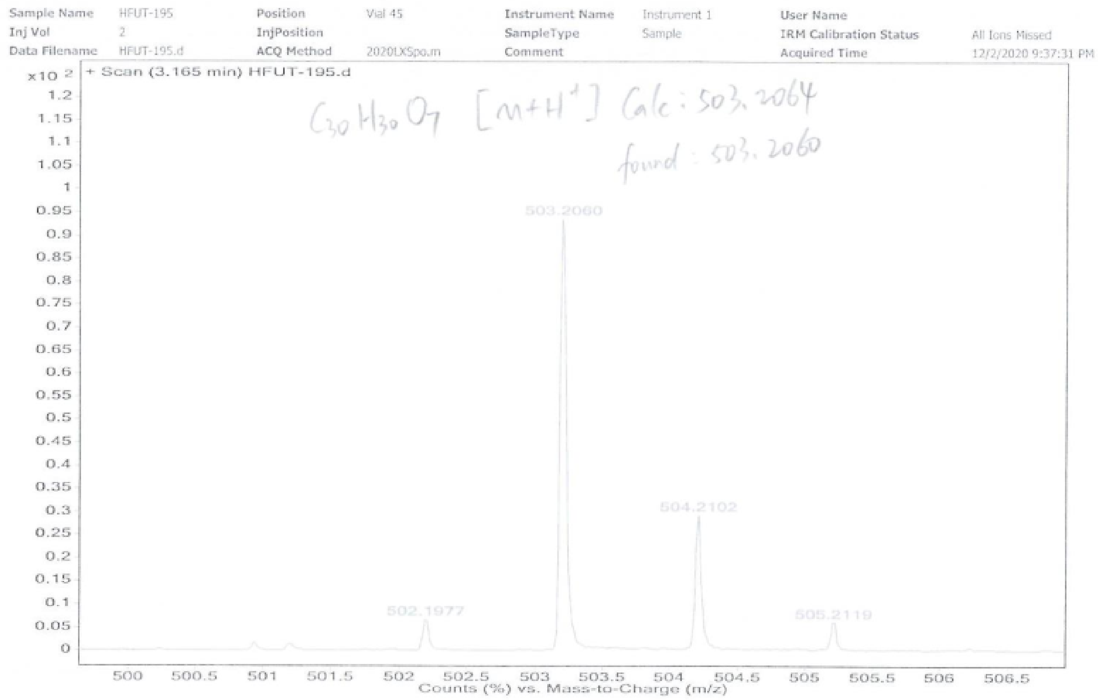

^{1}H NMR of compound **PIF_11**

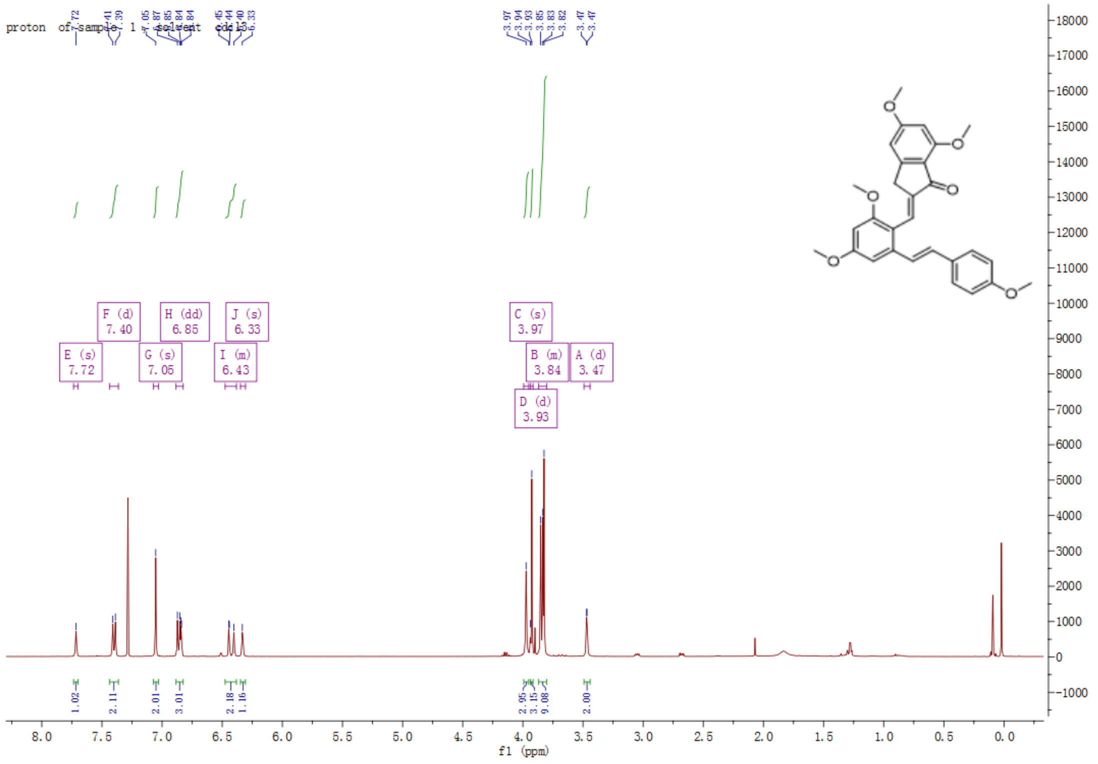

^{13}C NMR of compound **PIF_11**

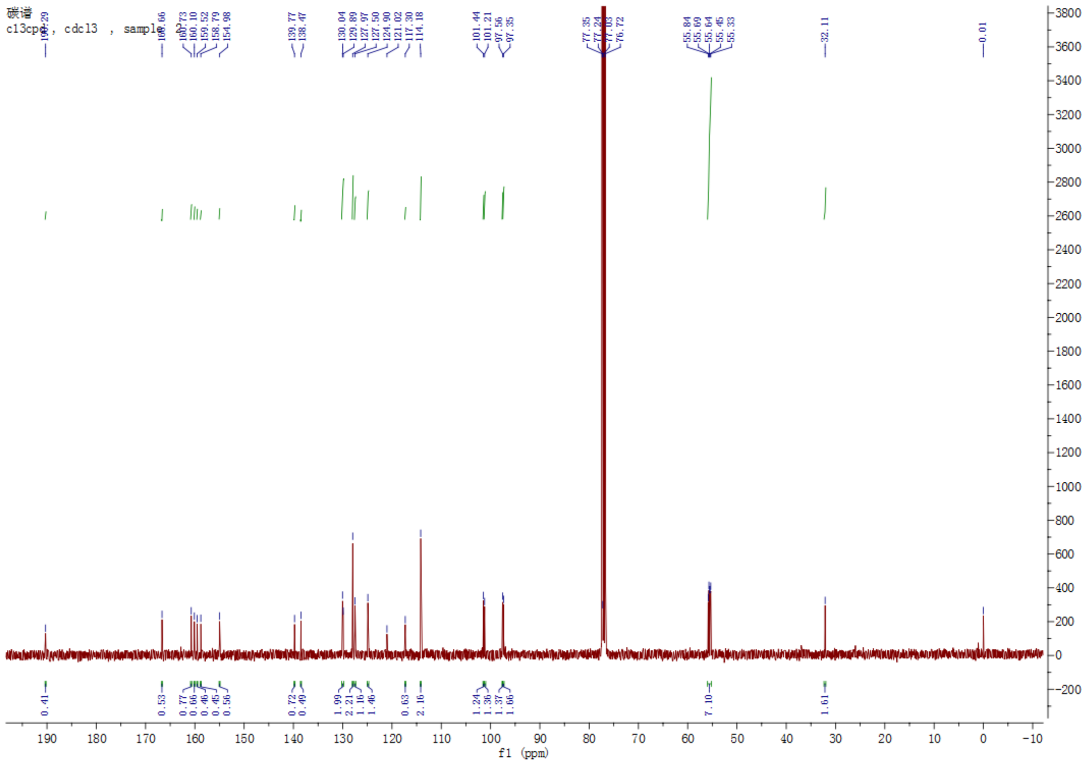

HR-MS of compound **PIF_11**

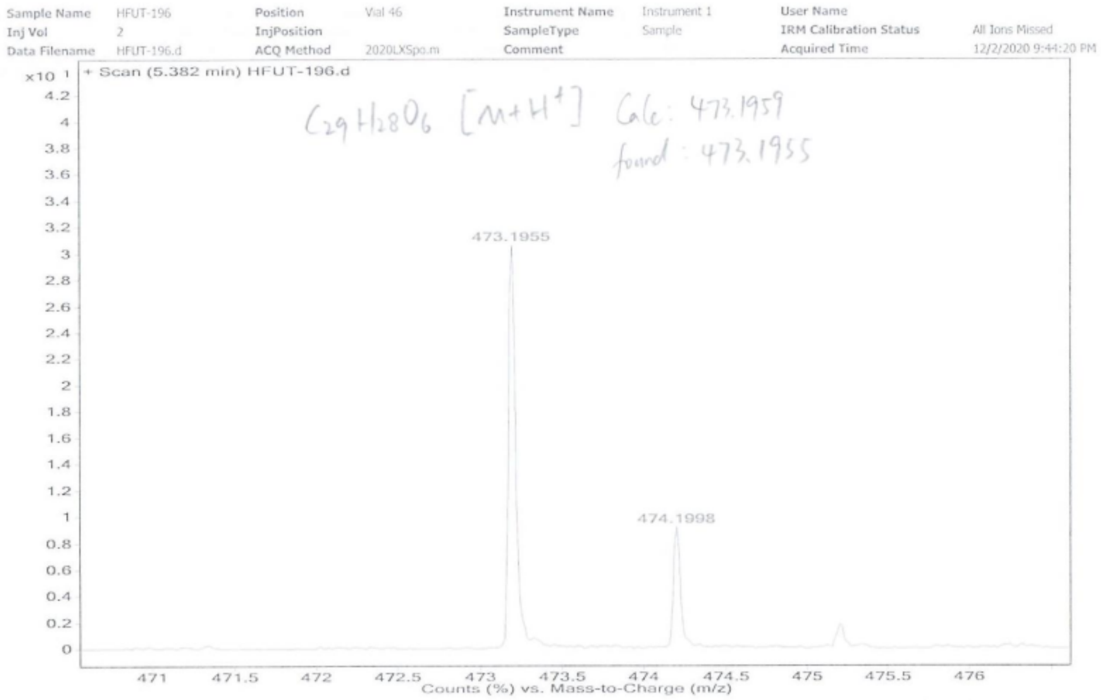

^{1}H NMR of compound **PIF_12**

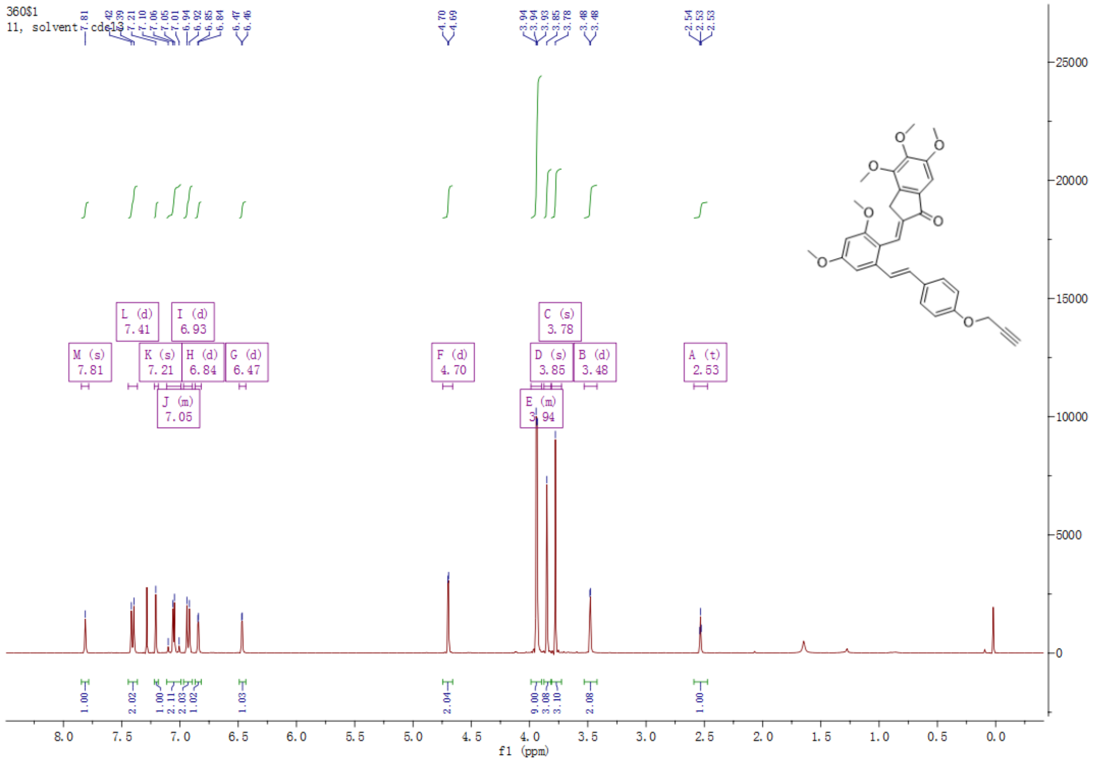

^{13}C NMR of compound **PIF_12**

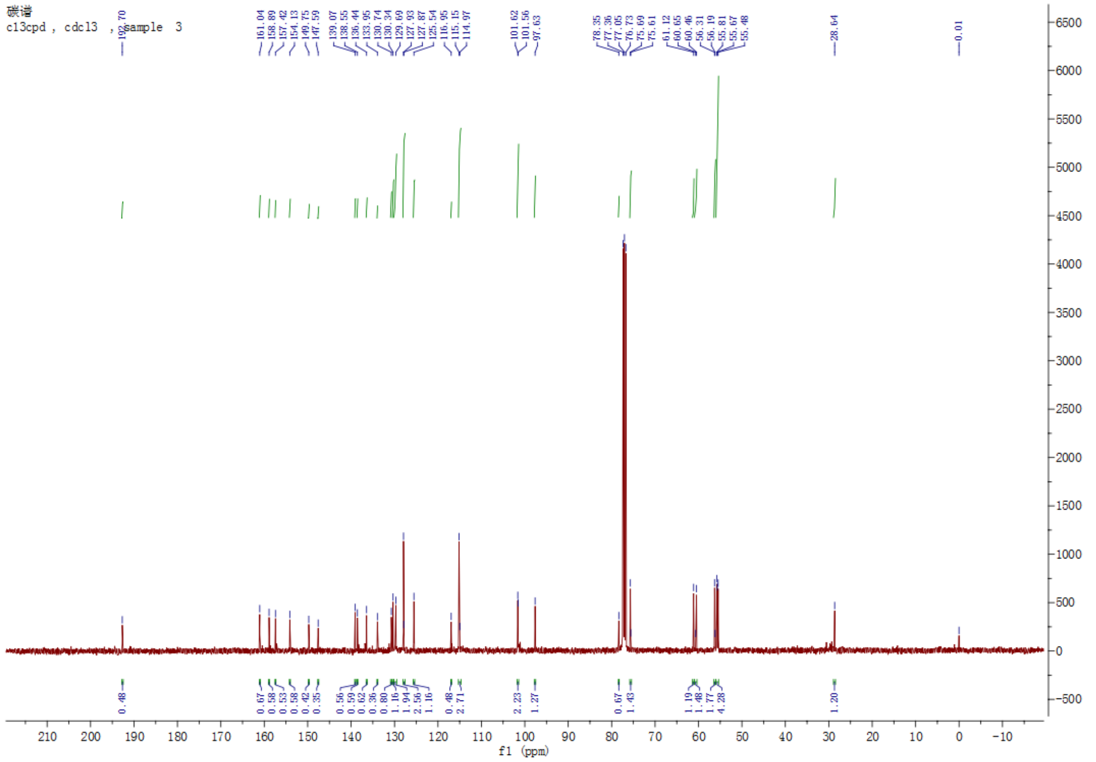

HR-MS of compound **PIF_12**

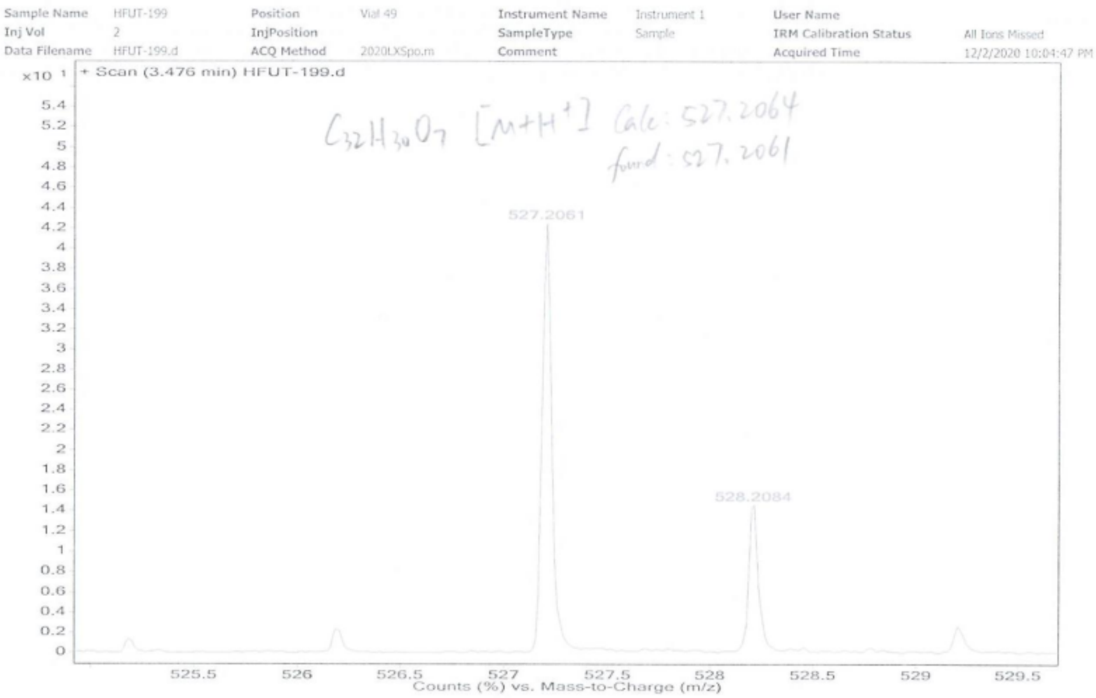

^{1}H NMR of compound **PIF_15**

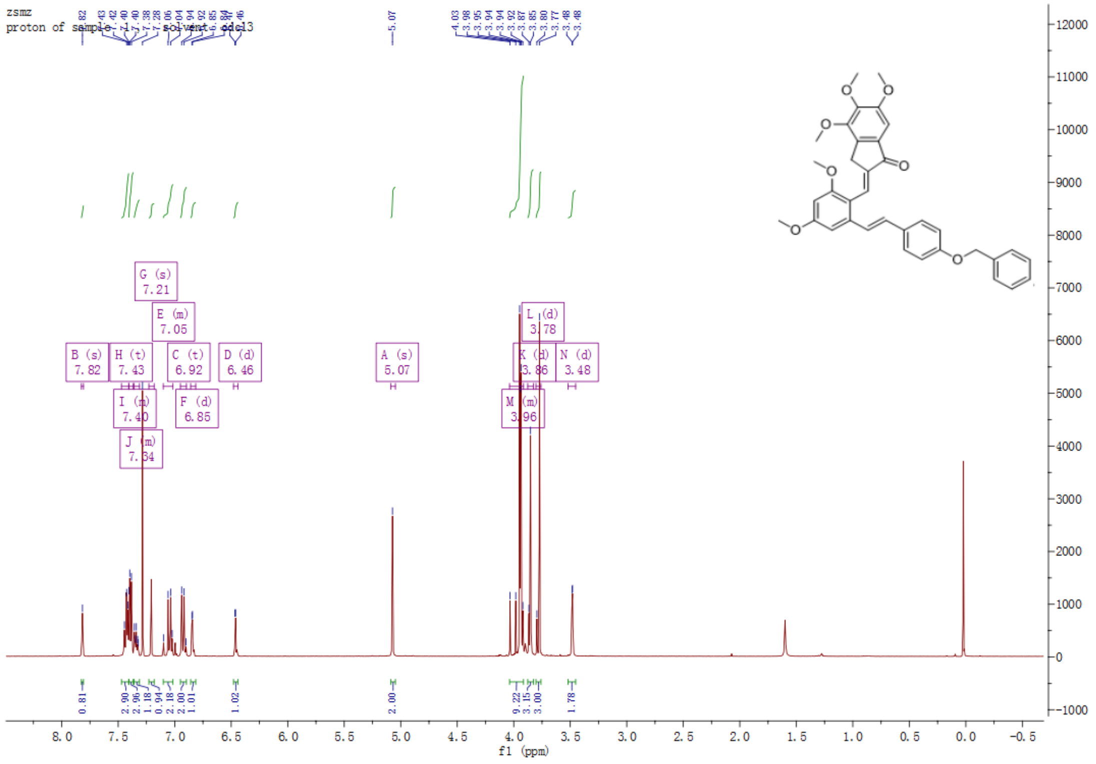

^{13}C NMR of compound **PIF_15**

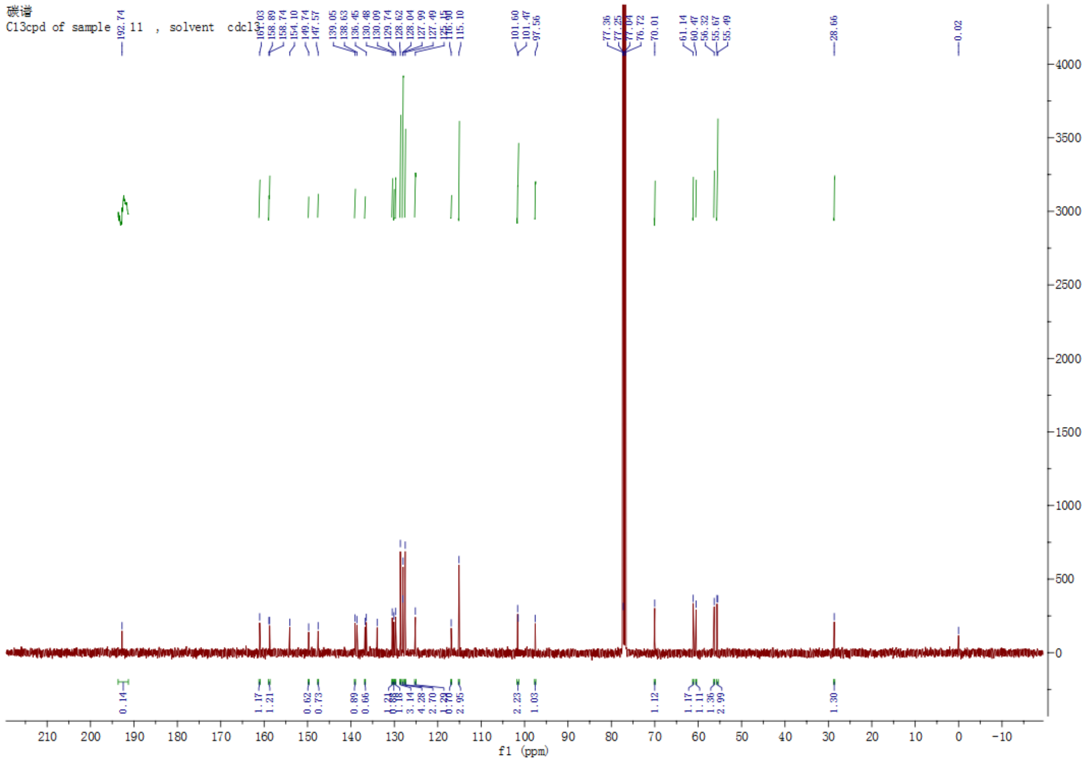

HR-MS of compound **PIF_15**

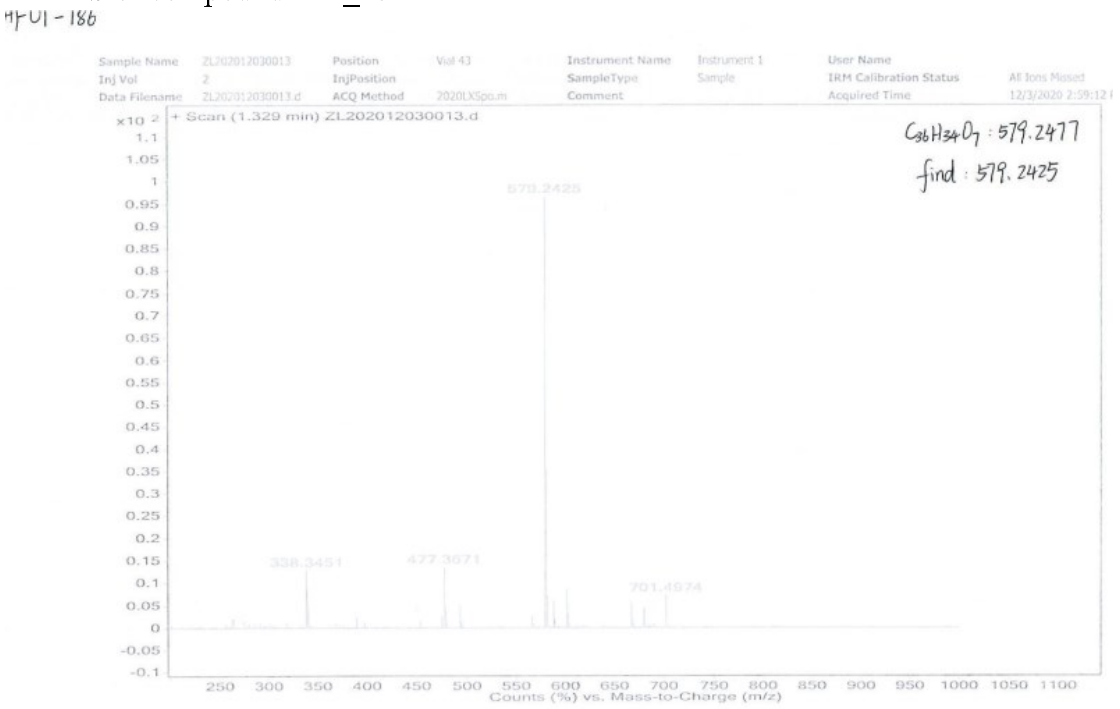

Supplement: Supplementary file 1 [file antioxidants-10-01333-s001.zip › antioxidants-1319885-supplementary.pdf]
